# The safety assessment of tampons: illustration of a comprehensive approach for four different products

**DOI:** 10.3389/frph.2023.1167868

**Published:** 2023-06-20

**Authors:** Anne E. Hochwalt, Joan M. Abbinante-Nissen, Lisa C. Bohman, Anne M. Hattersley, Ping Hu, Jan L. Streicher-Scott, Amber G. Teufel, Kara E. Woeller

**Affiliations:** ^1^ RWTS, LLC, West Chester, OH, United States; ^2^ Baby, Feminine and Family Care, Global Product Stewardship, The Procter & Gamble Company, Cincinnati, OH, United States; ^3^ Data Modeling and Sciences, The Procter & Gamble Company, Mason, OH, United States; ^4^ Global Safety Surveillance and Analysis, The Procter & Gamble Company, Mason, OH, United States; ^5^ Corporate Biosciences, The Procter & Gamble Company, Mason, OH, United States; ^6^ Baby, Feminine and Family Care Clinical Sciences, The Procter & Gamble Company, Cincinnati, OH, United States; ^7^ Baby, Feminine and Family Care Microbiology, The Procter & Gamble Company, Cincinnati, OH, United States

**Keywords:** tampon, safety assessment, biocompatibility, vaginal mucosa, vaginal microbiome, staphylococcal toxic shock syndrome, post marketing surveillance

## Abstract

**Introduction:**

We illustrate a comprehensive tampon safety assessment approach that assures products can be used safely. Material biocompatibility, vaginal mucosa assessment, vaginal microbiome evaluation, and *in vitro* assessment of potential risk of staphylococcal toxic shock syndrome expressed through growth of *Staphylococcus aureus* (*S. aureus*) and production of TSST-1 are the four essential portions of the approach. Post-marketing surveillance informs of possible health effects that warrant follow up. The approach meets or exceeds US and international regulatory guidance and is described through the example of four tampon products.

**Methods/Results:**

Each product is comprised mostly of large molecular weight components (cotton, rayon, polymers) that cannot pass the vaginal mucosa, are widely used across the industry, and replete with a vast body of safety data and a long history of safe use in the category. Quantitative risk assessment of all small molecular weight components assured a sufficient margin of safety supporting their use. Vaginal mucosa assessment confirmed that pressure points, rough edges and/or sharp contact points were absent. A randomized cross-over clinical trial (ClinicalTrials.gov Identifier: NCT03478371) revealed favorable comfort ratings, and few complaints of irritation, burning, stinging, or discomfort upon insertion, wear, and removal. Adverse events were few, mild in severity, self-limited and resolved without treatment. Vaginal microbiota assessment *in vitro* presented no adverse effect on microbial growth. Culture-independent microbiome analyses from vaginal swab samples obtained during the clinical trial showed no differences attributable to tampon usage, but instead due to statistically significant subject-to-subject variability. Growth of *S. aureus* and TSST-1 toxin production in the presence of any of the four products *in vitro* were statistically significantly reduced when compared to medium control alone*.*

**Discussion:**

The data from the four elements of the comprehensive safety assessment approach illustrated herein confirm that tampons evaluated using this system can be used safely for menstrual protection. A post-marketing surveillance system that monitors and responds to in-market experiences indicated in-use tolerability of the product among consumers, thus confirming the conclusions of the pre-marketing safety assessment.

## 1. Introduction

Tampons provide a convenient and effective form of menstrual protection. Over 100 million women in more than 120 countries use tampons for at least a portion of their menstrual protection. A 2022 Euromonitor report (1) stated that US women spent about $1 billion per year on tampons in the years 2016–2021. The percentage of US menstruating women who use tampons varies between 22% and 86%, depending on age and ethnicity. Tampon use is most prevalent among adolescents and young women: 71% of American adolescents and 81% of college students surveyed used tampons alone or in combination with pads (2–4). A survey of 1153 French consumers reports 45% of participants use tampons (5).

Assessing tampons to assure their safe use dates back as far as the 1940s (6, 7). Since then, new tampon designs or compositions have been accompanied by safety assessments (8–13). In the 1980s, women became acutely aware of Toxic Shock Syndrome (TSS), a rare but serious and potentially fatal illness that was linked to tampon use (14, 15). A detailed review of menstrual TSS (mTSS) can be found in Schlievert and Davis 2020 (16). In brief, TSS is caused by TSST-1-producing strains of *Staphylococcus aureus* that endogenously inhabit the vagina of some women (17). TSS risk increases with use of tampons; TSS also occurs with use of a variety of other vaginally-inserted products, including menstrual cups, cervical caps, diaphragms, pessaries, and natural sea-sponges (18–25). The illness occurs in men, boys, non-menstruating women, as well as menstruating women using pads (14, 26, 27). Because TSS risk increases with tampon use, assessing TSS risk is an important element of contemporary comprehensive tampon safety assessments.

After the TSS linkage to tampon use in the 1980s, authoritative bodies around the world set expectations that tampon manufacturers assure the safe use of these products. As an outcome, some authorities instituted guidelines for determining the safety of tampons. Examples include the US FDA Guidance for Industry and FDA Staff for premarket notification submissions for menstrual tampons and pads (28); the International Standard for Risk Management Application to Medical Devices: ISO 14971 (29), the principles of the General Product Safety Directive in the European Union (30); US FDA regulatory guidance for the use of ISO-10993, an international standard for biocompatibility testing (31); and the risk management principles outlined in the US Code of Federal Regulations 21CFR801.430 (32). The safety paradigm outlined herein is global in nature and meets or often exceeds these standards.

Tampon manufacturers provide women with safety information and recommended usage instructions in an appropriate language by regulation, or via compliance with industry codes of practices (32–34). Optimally, manufacturers will voluntarily provide this information in countries where no regulation or industry code of practice exists. In these regions, the voluntary inclusion of safety labelling further enhances women's safe use of tampons.

**Figure 1:**
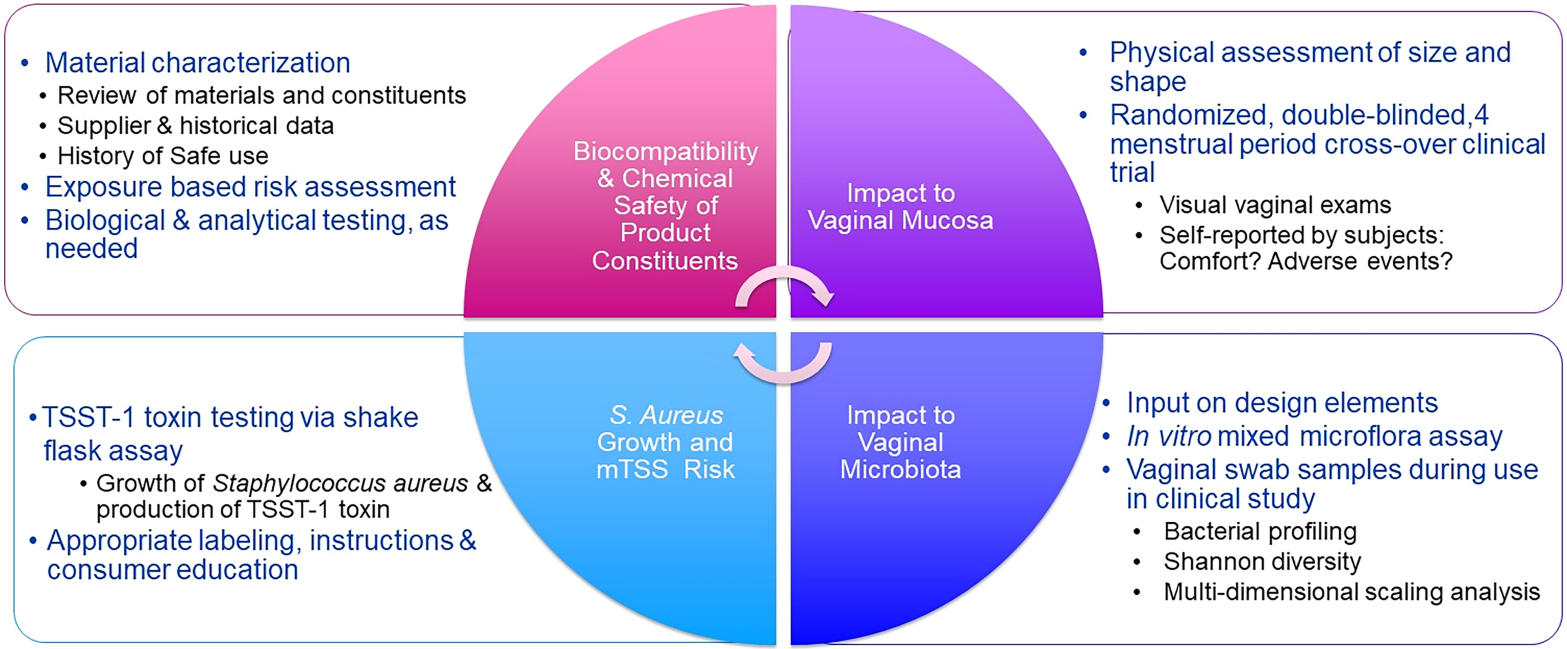
Tampon quadripartite safety scheme. Elements of a safety program are determined by the degree of product change. Not all elements are necessary for every change.

Because tampon safety communications have largely been conducted between the manufacturers and regulatory authorities, there has been a void in awareness and understanding about the rigor and extent of safety assessments completed to enable introduction of new products. This manuscript describes the comprehensive safety assessment approach that when applied, has assured authorities that tampons can be used safely. This four-part safety assessment approach was developed to assess the safety of Tampax and other P&G branded tampons (Figure 1) and is articulated through the example of four tampon products. The four-part approach includes biocompatibility and chemical safety of the product components; physical impacts to the vaginal mucosa; impact to vaginal microbiota; and risk for Toxic Shock Syndrome (TSS) and, as with all P&G products, safety surveillance. Other evaluations such as environmental safety and worker safety are not included in the scope of this manuscript.

It is important to note that a change in material or design does not in and of itself equate to or trigger testing. Testing may not be needed when there is sufficient scientific evidence supporting the safe use of the product. Conversely, a clinical trial or laboratory evaluation may be conducted if there has been an accumulation of minor modifications over a period of time that warrants confirmatory study. If and when testing is called for, the application of validated, up-to-date laboratory methods and a well-designed clinical protocol is imperative. The methods presented are provided as examples of suitable methods for generating specific data to support the overall safety assessment.

## 2. Materials and methods for a comprehensive safety assessment

A comprehensive safety assessment is completed in advance of a product's market introduction and may begin early in a new product design's development life cycle. The process is iterative, begins with the earliest prototypes and guides product development, assuring that use of the modified product (in consumer research, in clinical trials, or when marketed) is supported by sufficient safety data. Safety data from earlier prototypes can be used to inform subsequent safety evaluations of final designs intended for market introduction. Data gaps are identified and addressed appropriately.

### 2.1. Tampon product materials and design

A comprehensive safety assessment starts with disclosure of materials and material constituents. Suppliers provide proprietary disclosures of their materials identifying their individual constituents, material processing information (e.g., processing aides, fiber purification processes), and likely impurities from the manufacture, processing, or sourcing of the materials. Some materials may have more than one constituent, and constituents may have more than one sub-constituent, all of which are disclosed, throughout various layers of the supply chain. Novel shape, new structures (i.e., absorbent braid), design elements (i.e., layered vs. blended fibers) and manufacturing aids of the tampon and applicator are also included in the safety assessment.

**Figure 2:**
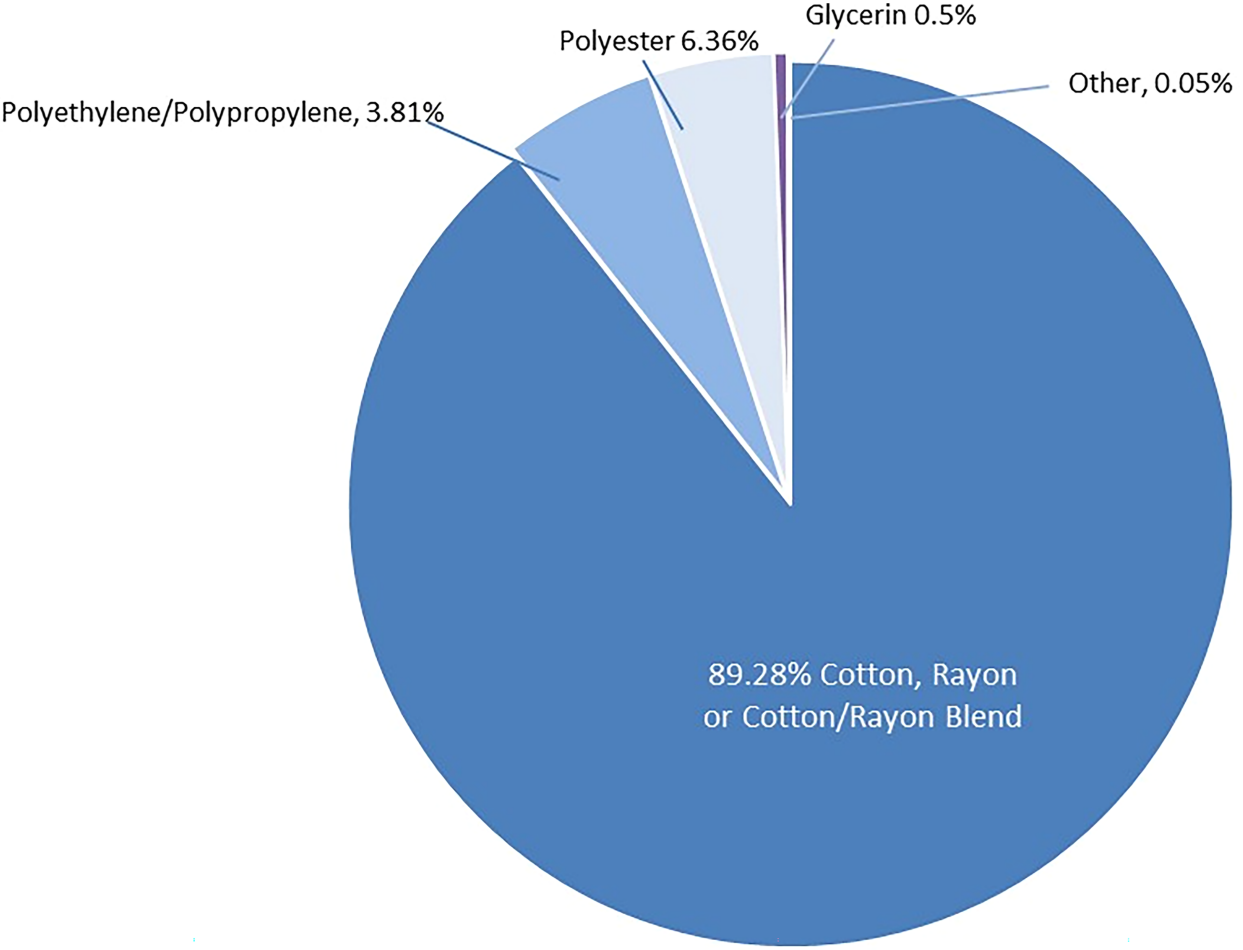
Test product composition.

The materials and design of the four tampons assessed in this paper are described in Table 1. These products are Procter & Gamble Co. Tampax Pocket Pearl (TPP), Tampax Pure Cotton (TPC), Tampax Compak (TC), and Tampax (with a cardboard applicator) (TCA). The composition of these tampons is similar to tampons of all manufacturers (35, 36). Tampon design and composition has remained remarkably consistent over the past several decades and thus carries a long history of safe use and experience. Like most other feminine hygiene products (pads, liners, adult incontinence products) on the market, the study tampons are largely comprised of large molecular weight polymers (Figure 2). The absorbent fibers are cotton, rayon or a blend thereof (cotton and rayon are cellulosic polymers); overwraps, removal cords, sewing threads (that attach the removal cord to the tampon), and secondary absorbent materials are cotton, rayon, polypropylene, polyethylene, and/or polyester. The remaining components are present to enable proper fluid management or enable opacity. Tampax tampons are inserted into the vagina with a tube-shaped applicator comprised of plastic or cardboard. The product is enclosed in a wrapper to enable sanitary handling of the product before it is inserted into the body.

**Table 1:** Study product composition [regular absorbency (6–9 g)].

	TC	TCA	TPP	TPC
Absorbent Fiber	Rayon	Rayon	Rayon/Cotton	Cotton
Sewing Thread	Cotton, Polyester	Cotton	Polyester	Polyester
Removal Cord	Polyester	Polyester	Polypropylene/Cotton	Polypropylene/Cotton
Overwrap	Rayon, Polyester	Rayon, Polyester	Polypropylene/Polyethylene	Cotton
Secondary Absorbent	Rayon, Polyester	Rayon, Polyester	Polypropylene	Polypropylene
Applicator	Plastic	Cardboard	Plastic	Plastic
Other*	Paraffin	Paraffin	Glycerin, Ethoxylated Fatty Acid Esters, PEG-100 Stearate, Paraffin	Glycerin, Paraffin

* Other: also includes other intentionally added materials (<0.55%) that provide a function for fluid handling and absorbency within the product.

### 2.2. Raw material biocompatibility

Raw material biocompatibility is the foundation of the tampon safety assessment. The paucity of published literature summarizing the biocompatibility of tampon raw materials belies the volume of data informing their long history of safe use. The 1976 US FDA Medical Device Amendment to the Federal Food, Drug, and Cosmetic Act and the subsequent 1980 Final rule classifying tampons as Class II medical devices formalized the systematic documentation of tampon material safety. The Amendment and Final Rule ensured regulation of all materials used in tampon manufacture; the Class II medical device designation codified biocompatibility assessments and material assessments as necessary to reduce risks to consumers. Today, the International Standard ISO 10993-1 “Biological evaluation of medical devices-Part 1: Evaluation and testing within a risk management process” guides both regulatory authorities (31) and manufacturers who sell products in the US to assure the biocompatibility between the device materials and the body. The comprehensive safety assessment described herein meets or exceeds the expectations of this regulatory framework.

Quantitative risk assessment (QRA) principles ascertain material safety; QRA as articulated by the National Academy of Sciences (37–39) is recommended. This interdisciplinary process, also applied to safety assessment of other consumer products (40–45), hinges on gaining an evidence-based scientific understanding of potential toxicological hazards in the context of the relevant human exposures (dose, route and duration) in order to quantitatively estimate the risks associated with such exposures over a menstrual lifetime. All relevant endpoints including systemic toxicity (repeat-dose organ or general toxicity, reproductive and developmental toxicity, genetic toxicity, and carcinogenicity) and local effects (including mucosal irritation and contact sensitization) are considered. Where there are toxicology data gaps, the principle of “Structure Activity Relationship” (SAR) or “Toxicological Threshold of Concern” (TTC) can be used to establish an exposure limit below which there is not concern for human safety (31, 46–50).

Because the vast majority of a tampon (by weight) is comprised of cotton or rayon and other large molecular weight polymers that are inert and unable to cross through the vaginal mucosa, chemical assessments focus on the smaller molecular weight substances (e.g., processing aid residues, fiber treatments, impurities) that could be available for absorption and could potentially cross the vaginal mucosa, depending upon the chemical and/or physical properties of the particular substance and its use in the finished product. The initial estimates of consumer exposure from product use are based on conservative, default exposure assumptions (Table 2). If the initial conservative estimate results in a sufficient margin of safety (MOS > 1), no further refinements are needed. The MOS is the chemical constituent reference dose [RfD: defined as an estimate of a daily exposure to the human population (including sensitive subpopulations) that is likely to be without an appreciable risk of deleterious effects during a lifetime of exposure] with incorporated uncertainty factors divided by the consumer exposure, which should be greater than one for positive assurance of safety.

**Table 2:** Default values^a^ for initial screening assessments.

Description	Abbreviation	Default Value and Unit (Unless Noted Otherwise)
Product component weight (g)/tampon	RMW	X g/tampon (Actual component RMW used)
Concentration of constituent in product component	Cr	Y % (g of constituent/100 g of tampon)
Frequency of use	F	5 tampons/day^b^
Transfer factor	T	100%
Conversion factor (g to µg)	Cf	1,000,000 µg/g
Mucosal absorption	Ab	100%
Exposure duration	Et	100% (daily lifetime exposure; ∼70 years)
Body weight	BW	50 kg^c^
Surface area of tampon	SA	Z cm^2^ (allergic contact sensitization assessments)
**Calculation of Default total exposure for continuous, direct mucosal contact constituents**		
Systemic exposure (µg/kg/day) = (RMW × Cr × F × T × Cf × Ab × Et)/BW		
Local exposure (µg/cm^2^/day) = (RMW × Cr × F × Cf × Et)/SA		

^a^ Default values may be refined with further study and with substance-specific data, or specific duration of tampon wear for example.

^b^ Internal habits and practices data, unpublished.

^c^ Representative of lower end of body weight reported for teen girls.

Should the initial results identify substances for which there is an insufficient MOS (MOS < 1), additional specific scientific evidence enables refinement of the parameters used to calculate the MOS. This may include targeted analyses to refine exposure based on substance-specific analytical data using physiologically relevant conditions [similar to the extractables and leachables approach used in food packaging contact regulation (51)]. Surrogate exposure models that mimic consumer use can be employed to obtain more realistic consumer exposure values. Lastly, redesign of the product, reduction of the amount of constituent under evaluation, or elimination of the substance are other options to consider for substances with inadequate MOS. Only when there is a sufficient MOS for the substance can it be approved for use in product. A brief summary of historic biocompatibility studies conducted with cotton and rayon fibers as well as tampon finished products are presented in Table 3A. These data offer additional confidence of the safety of these materials.

**Table 3A:** Historic (unpublished) Tampax biocompatibility, vaginal mucosa, vaginal microbiome and TSST-1 toxin and *S. aureus* growth data.

Study	Objective	No of studies	Test materials	Protocol, guideline and/or reference	Results	Conclusion
Cytotoxicity Assay	To evaluate the cytotoxicity potential using an *in vitro* mammalian cell (murine L 929 cells) culture test.	54	Applicators, whole tampons (cotton, rayon, or blended cotton/rayon fiber) or tampon components extracted in saline (1 g/5 ml, 20%) cotton seed oil/media double extract (*n*-153 materials tested)	USP, ISO 10993-5 compliant (52) Extraction conditions: 37°C for 24 h)	Reactivity grades: 0 (reactivity = none) to 1 (slight reactivity) for all test materials. Study considered valid in all cases with positive controls yielding ≥ grade 3 (moderate reactivity) and the negative control yielding a grade 0 in all cases.	Minimal (∼20% of studies) to no (∼80% of studies) evidence of cytotoxicity
Ames Assay	To evaluate a chemical's genotoxicity by measuring its ability to induce reverse mutations at selected loci of several bacterial strains	4	Tampon components (e.g., fragrance) and cotton or rayon fiber extracted in saline with and without metabolic activation	Ames et al. (53) and Maron, Ames (54) Or OECD 471 (55)	Test materials did not cause a positive response either in the presence or absence of liver microsome activation system	No evidence of reverse mutations
Mutation Assay	To evaluate the potential to induce forward mutations at the Hypoxanthine-guanine phosphoribosyl transferase (HGPRT) locus of Chinese Hamster Ovary cells	3	Tampon components (e.g., fragrance) studies of cotton or rayon fiber in saline extract	OECD 476 (56)	None of the treated cultures exhibited mutant frequencies greater than 40 mutants per 10^7^ clonable cells	No evidence of forward mutations with or without S9-activated systems;
HRIPT	To evaluate the potential to induce irritation or allergic contact dermatitis in (at least 100) human subjects	12	Tampon components, extracts of tampon components or saline moistened fiber	Gerberick et al. (57, 58)	None of the subjects displayed a pattern of dermal reactions indicative of an allergic response. Additionally, no significant cumulative irritation was seen with tested materials.	No irritation or allergic contact dermatitis

**Table 3B:** Historic (unpublished) tampax vaginal mucosa, vaginal microbiome and TSST-1 toxin and *S. aureus* growth data.

Study	Objective	No of Studies	Test Materials	Protocol, Guideline, and/or Reference	Results	Conclusion
*In vitro* TSST-1 Toxin Testing	To evaluate the device for its effect on bacterial growth and TSST-1 production *in vitro*	69	Applicators, whole tampons (cotton, rayon, or blended cotton/rayon fibers or tampon components *N* = 213 test materials studied.	Parsonnet (59), Schlievert (60)	TSST-1 Toxin product, S. aureus count, and Toxin product/cfu	No adverse effect on S. aureus growth or TSST-1 toxin product
*In vitro* microflora assessment	To determine if the device had a bactericidal or bacteriostatic effect on microorganisms selected to be representative bacterial strains of the vagina	13	Whole tampons (cotton, rayon, or blended cotton/rayon fiber) or tampon components; over 50 materials tested	Testing with a mono-culture: Unpublished method by AB Onderdonk Testing with a consortium of organisms: Sica et al. (61)	Effect on organism growth and cell density	No adverse effects on growth of sentinel organisms when tested individually or in a consortium
In-use Clinical Safety Studies assessing vaginal condition	To assess vaginal condition before and after tampon use	15	Cotton, rayon, or blended cotton/rayon tampons	Use product as normally would; experience diaries; tissue examination	>1,700 subjects, and >85,000 tampons with only sporadic reports of vaginal irritation	No irritation nor adverse vaginal condition effects attributed to use of products
In-use clinical microflora assessment	To assess impact of the device on vaginal microorganisms (culture based-methods)	11	Rayon, cotton, or blended rayon/cotton tampons	Use product as normally would, except on sample day-wear 6 h; tampon, vaginal swabs (intra and intermenstrual)	Tampons, swabs from >100 study subjects assessed for presence and cell count of keystone microorganisms	Regardless of sample composition, organism presence within expected ranges and cell counts within ranges established for vaginal microflora

### 2.3. Vaginal Mucosa assessment

Physical effects attributed to the intimate contact of the tampon with the vaginal mucosal tissue are uncommon. Regardless, assurance of no trauma attributable to the new or modified tampon or applicator remains important. Published reports are rare and show occurrence of cervicovaginal dryness, microtrauma, abrasion, and ulceration (62, 63). No clinical significance has been attributed to any of these occurrences.

Several publications address the in-use clinical assessment of tampons being newly introduced into the market (10–13, 64). Additional unpublished Tampax clinical in-use studies (Table 3B) assessing >1,700 study subjects and >850,000 tampons show occurrence of objective vaginal effects has been virtually nil and limited to sporadic erythema or rare complaints of discomfort. While unpublished, these studies demonstrate the history of evaluations and the safe use of these intravaginal products.

The clinical significance of any visual observations must be carefully considered, as some alterations are unrelated to product use but may represent normal variability in the state of the epithelium (65). For example, superficial epithelial changes unrelated to tampon use have been observed over four to six months of colposcopic inspection of the vaginal epithelium of healthy women (62). Advancing age/perimenopause, smoking, intercourse in the prior 72 h and other likely confounding factors (the menstrual cycle, certain disease states, medications, exogenous hormones, barrier contraceptive use) may create background “noise” of uncertain clinical significance (62, 66). These factors should be considered when designing and evaluating clinical studies with vaginal exam endpoints.

#### 2.3.1. Visual assessment of four tampons products

Tampons and applicators are to be routinely examined visually and by touch in a safety assessment to ensure pressure points, rough edges and/or sharp contact points are absent.

#### 2.3.2. Randomized clinical trial of four tampons

Further assurance of no adverse physical effects on the vaginal tissues may include objective vaginal tissue examination and subjective sensory assessment during a prospective clinical trial as described below. Subject assessment often includes product experience diaries completed by study subjects that enable capture of subjective sensory feedback that complement the more objective visual assessments. Diaries may be completed with every tampon change and at the end of every menstrual cycle. Diaries may capture tampon wear time, subjective irritation (for example, burn, sting, itch during product wear, as well as comfort during tampon insertion, wear, and removal) and general subject comfort data. Diaries and questionnaires may also capture an overall comfort or satisfaction assessment with the product at the end of each menstrual cycle.

A double-blinded, 4-period cross-over clinical trial evaluating the four tampon products for safety and tolerability via vaginal health endpoints addressed vaginal mucosal safety and tolerability. The study was reviewed and approved by Integreview IRB, Austin Texas (IRB#: 00001035). Study procedures were monitored by a representative of the Sponsor according to the US CFR Title 21 Part 312 and ICH Guideline for GCP (Section 5.18). Generally healthy women aged 18–55 who reported having consistent menstrual cycles and who typically used tampons as their main source of feminine protection during menstruation were enrolled after signing a study-specific informed consent form. During the enrollment visit, menstruating women were screened against defined inclusion and exclusion criteria, tested to be free from sexually transmitted and vaginal infections (gonorrhea, chlamydia, trichomonas, bacterial vaginosis, and yeast), using an effective form of birth control, and confirmed not pregnant (urine testing).

Subjects who met the enrollment criteria were given one of 4 regular absorbency tampon products to use during their next menstrual cycle assigned by randomization to treatment groups. The subjects and Investigator were blinded; products were not identified by product type, but they were not identical in appearance. One randomized product was tested per consecutive monthly menstrual cycle over the course of the study. The subjects were given a diary to be completed after each tampon use and a monthly Comfort Questionnaire to better understand product-related experiences on subject-reported outcomes related to sensorial experiences and comfort. Women who chose to use a backup menstrual protection product in addition to their assigned study tampon were provided currently marketed Always menstrual pads and liners for use as needed throughout the study.

Subjects were screened at enrollment to exclude subjects with positive results for BV (Bacterial Vaginosis), *Candida spp., Trichomonas vaginalis*, *Chlamydia trachomatis,* and *Neisseria gonorrhoeae*. Subjects were given instructions to refrain from using other vaginal and vulvar products, such as antibacterial soaps, lotions, powders, pubic hair removal and to refrain from sexual activity for at least 48 h prior to every gynecological examination visit. Randomized subjects were directed to use the study tampons as they normally would use their own tampons. During the tampon use phase of the study, subjects were queried for changes in their health and medications at every visit.

Subjects returned to the site within 72 h of their last tampon use for an assessment of vaginal health conducted by the site medical Principal Investigator (PI) physician using a lighted speculum. Erythema was graded at 6 different sites (labia minora, introitus, lower vaginal wall, middle vaginal wall, upper vaginal wall including fornices, and the cervix) using a scale of 0–4 with 0 being no erythema and 4 being severe erythema. The presence or absence of vaginal abrasion and ulcerations were noted at each of the 6 grading sites. Subjects were asked about health and compliance at each visit. Adverse events (AEs) reported by the subject, observed by study personnel or from any laboratory tests were assessed throughout the study as another measure of safety and tolerability. A swab sample for microbiome analysis was also collected at this visit.

The sample size of 65 subjects (the minimum number needed to complete the study) was determined using clinical results based on expert judgment related to relevant publications/past clinical studies. A larger base size was enrolled to compensate for anticipated dropouts over the course of the study. Prior to statistical analysis, all data were checked for accuracy, completeness, and compliance with the protocol. Analyses were completed using PC SAS Release 9.4. Demographic data were summarized in tables of descriptive statistics by treatment sequence to assess overall balance between assigned sequences. Summary descriptive statistics were completed for all parameters. Confidence intervals (95% confidence intervals) were constructed for all clinical assessments for each treatment code. Any data reported as “unable to evaluate” were treated as missing and excluded from the analysis.

### 2.4. Vaginal Microbiota assessment

The microbiota inhabiting the healthy vagina include symbiotic and commensal organisms that modulate vaginal physiology, maintain a healthy vaginal pH, and compete against indigenous species or invading microbes that can cause disease. Assuring microbial growth remains unaffected in the presence of the tampon can be assessed in an *in vitro* mixed microflora assay by exposing the tampon to a consortium of organisms containing predominant genera such as *Lactobacillus* and organisms that may give rise to gynaecological diseases or infection such as bacterial vaginosis, pelvic inflammatory disease, urogenital infection, vaginal yeast infection, and Toxic Shock Syndrome. Historic *in vitro* data (Table 3B) repeatedly and consistently assured that microbial growth remained unaffected in the presence of the tampons.

Culture-dependent or culture-independent microbiome analyses of vaginal fluid or tampon samples from women who participate in prospective trials provides further means to assess test products and reference controls with a history of safe use (10–13, 64, 67). Samples are assessed for presence and cell density of organisms and are compared to baseline as well as to other product usage. Other pertinent outcome measures in such trials may include vaginal pH which, in healthy women, is slightly acidic between menstrual cycles and nearer to neutral during menstruation (68) and evaluation of the color and consistency of vaginal discharge, which can be a sign of infection. With culture-dependent analyses, evaluation of microbes (such as *Lactobacillus*, which support a healthy vaginal ecosystem), as well as evaluation of gynecologically important organisms which can change with life stage or can be give rise to gynecological diseases or infection may also be individually assessed. The absence of significant microbiological risk from the product is supported when no biologically significant difference is observed in clinical isolation patterns of vaginal microbes at clinically meaningful levels (typically characterized as change of 2 log or greater) when compared to reference controls (69). The 2 log criterion was based on work by Onderdonk and colleagues (69, 70) who determined that variations of mean aerobic and anaerobic counts could range from 1 to 2 log CFU/g without any negative impact on health. In addition to the available published literature, 11 unpublished Tampax vaginal microbiome studies (Table 3B) provide historic perspective and underscores the absence of observed adverse effects on the presence or cell density of vaginal microbes after 1 or more menstrual cycles.

Culture-independent methods based on 16S rRNA gene sequences can be used to overcome limitations of culture-dependent methods. These methods reveal the phylogenetic diversity of microorganisms present in the vaginal microbiome, not possible with culture-based methods. Vaginal microbiome studies to-date confirm the significant diversity of normal vaginal communities (67, 71–75).

#### 2.4.1. *In vitro* assessment of four tampons on representative vaginal microbiota

An *in vitro* mixed microflora assay previously described in detail (61) served to determine if 48-hour exposure to the four study tampons had a bactericidal or bacteriostatic effect on a consortium of six microorganisms. This assay was originally developed in collaboration between Procter & Gamble and Microbiologists Specialists, Inc. (Houston TX, USA) (76) since standardised test methods addressing the potential impact of intravaginal feminine hygiene articles on the vaginal microbiome are unavailable. The six microorganisms included in the *in vitro* mixed microflora assay (*Lactobacillus gasseri, Gardnerella vaginalis, Prevotella bivia, Escherichia coli, Staphylococcus aureus, Candida albicans*) were selected to simulate the heterogenous nature of the healthy vaginal microbiome while also including organisms that might give rise to infection or disease (77).

The *in vitro* mixed microflora assay was conducted by Advanced Testing Laboratory, Inc., Cincinnati OH (USA). Briefly, the *in vitro* mixed microflora assay includes a preparatory stage, i.e., the preparation of freezer stocks and confirmatory identification of each organism, followed by three experimental stages, i.e., (I) the preparation of the stock inoculum by plating, incubation of individual organisms on specified media, and measurement of cell densities; (II) co-inoculation of each control and test product followed by incubation under anaerobic conditions; (III) plating of organisms on selective media following sample collection from control and test products.

After 48 h of selective growth (24 h for *E. coli*), the colony count of each test flask and control flask were recorded and adjusted by the dilution factor to determine the numbers of colony forming units (CFU/ml). All CFU counts were log normalized (log10) to facilitate data comparisons. Differences were calculated as the absolute deviation from the mean CFU (log10) for the product-containing flasks and those of the negative control. A perturbation of the microflora by ≥2 log change from the microorganism-containing negative control (consortia only) after 48-hour test product exposure was defined as indicating failure of the test product in meeting microbial safety requirements. The 2-log criterion was based on work by Onderdonk and colleagues (69, 70) who determined that variations of mean aerobic and anaerobic counts could range from 1 to 2 log CFU/g without any negative impact on health.

#### 2.4.2. Culture-Independent microbiome analyses of vaginal fluid

Vaginal swab samples were obtained from subjects participating in the 4-tampon in-use clinical study described above, at baseline and after use of each product for microbiome analyses. In addition, vaginal pH via pH paper was measured, and vaginal discharge assessed as normal or abnormal by medical Principal Investigator (PI) within 72 h of their last tampon use. If discharge was abnormal, color and consistency were evaluated. Vaginal pH changes and changes in vaginal discharge can be markers of vaginal infection or dysbiosis.

While the vaginal microbiome is relatively stable, factors like menstrual cycle, pregnancy, use of contraceptives or antibiotics and diet can affect the composition (78–81). Therefore, subjects were asked to use at least one form of birth control and were excluded from study participation for recent antibiotic or antifungal use. Further, throughout the trial, subjects were asked to refrain from use of antibacterial soap and/or any vaginal/perianal product, as well as from genital hair removal. Also, subjects were asked to refrain from vaginal intercourse for 48 h prior to the visit at which the swabs were taken, and to refrain from bathing within 24 h and showering within 12 h of the visit.

An initial analysis of the vaginal microbiota was conducted by Rocio Navarro Garcia, Research and Testing Laboratories (RTL) Genomics (Lubbock, TX, USA) and the final analysis by the authors of this study. The data analysis methodology followed that described by RTL Genomics (82) and Teufel and colleagues (83). In brief, amplicons of the target regions of the 16S rRNA genes were produced by polymerase chain reaction (PCR), followed by determination and classification of the gene sequences of the amplicons as described previously (61). The 16S sequences were done in two different batches. First batch includes samples from Baseline, TPP, TPC, the second batch included samples from TC and TCA. Sequences universally present in one batch but not in the other batch were excluded to eliminate the batch effect.

Various statistical tests were performed to determine changes within the vaginal microbiota, and if these changes were attributed to use of a specific tampon or if they were attributed to subject-to-subject differences. Both the paired Wilcoxon Rank Sum test (nonparametric) and paired student t-test (parametric) were performed to determine if microbial components differed between each pair of treatment groups. Kruskal Wallis tests detected microbial components differences among any of the four treatment groups. FDR-adjusted *p*-values (using Benjamini-Hochberg adjustment) were reported to limit the false positive result. It is important to note that the sequencing was completed in two separate runs at different time points due to availability and timing of samples and sequencing availability. For this reason, special care was taken in all data analysis to identify possible batch effects.

Shannon diversity (84, 85) was reported as the alpha diversity measurement (quantifying the microbial diversity of a sample). Multi-Dimensional Scaling (MDS) calculated using Bray-Curtis similarities (86) served to evaluate beta diversity (87) (measuring the similarity or differences between samples) i.e.,:

1. How each sampling time point (i.e., baseline and after use of each of the 4 tampons) compared against the other sampling points, and
2. How each subject's 5 recordings (baseline and after use of each of 4 tampons) compared against the 5 recordings for all other subjects.

Paired Wilcoxon Rank Sum tests, paired student T-tests and Kruskal Wallis tests were used to detect alpha diversity differences. Adonis tests (88) were performed to test beta diversity differences among different groups either based on treatment, sampling time points or individual subjects. Thus, these statistical methods determined if changes of the vaginal microbiota were predominantly caused by use of a specific tampon, or if they were rather accountable to inter-individual differences.

### 2.5. *S. aureus* Growth and TSST-1 Toxin Production

A critical health concern associated with the use of tampons is menstrual Toxic Shock Syndrome (TSS), a serious, but rare, recognizable and treatable disease caused by toxigenic strains of *S. aureus* that endogenously inhabit the vagina of some women (14, 15, 17, 89). Virtually all cases of menstrual TSS are associated with the superantigen, TSST-1 (90–94). Menstrual TSS is rare; incidence is reported to be ∼1 case/100,000 menstruating women and has remained unchanged since the 1980s (95–101). For this disease to occur, a woman must be vaginally colonized with a toxigenic strain of *S. aureus* and also must lack sufficient antibodies to the TSST-1 toxin. Moreover, conditions must exist such that TSST-1 is produced and transported across the mucosal membrane into the underlying tissue to result in systemic disease (16). The prevalence of vaginal colonization in normal healthy women with toxigenic strains of *S. aureus* ranges from 1%–3% (17, 102, 103).

A variety of methods have been used to examine tampon effects on *S. aureus* growth and TSST-1 production (59, 60, 104–107). Tampons should not promote TSST-production or *S. aureus* growth, as demonstrated by statistical comparison to medium control and in-market control products with an established safety profile. An abundance of historic TSST-1 toxin production assays (Table 3B) has been conducted with Tampax tampons initially using the Parsonnet shake flask method (59) and more recently, the Schlievert shake flask method (60) which utilizes similar methodology with minor modifications to that of the Parsonnet method. These data repeatedly and consistently confirm that the composition of these products affected neither *S. aureus* cell count nor TSST-1 toxin production, under these test conditions.

#### 2.5.1. *In vitro* assessment of four tampons on *S. aureus* growth and TSST-1 Toxin production

The effect of the four Tampax tampons on growth of *S. aureus* MN8 (a strain isolated from the vagina of a TSS patient and known to produce a high concentration of TSST-1 toxin) and production of TSST-1 was assessed by P. M. Schlievert, Department of Microbiology and Immunology, Carver College of Medicine, University of Iowa, Iowa City, IA (USA). The applied methodology follows that described by Schlievert and Blomster with further details on its application to assess intravaginal menstrual and contraceptive products (108). In brief, the method includes exposing the test article to cultures of 10^7^ *S. aureus*/ml of Todd Hewitt broth. After 18 h, the supernatants, which will contain any TSST-1 produced, are collected and serially diluted. These dilutions of the TSST-1 preparations are reacted against antisera (produced by the hyper-immunization of rabbits) in Ouchterlony immunodiffusion assays (109) to establish a toxin titer (TSST-1 µg/ml). TSST-1 concentrations were also determined by Western immunoblot analysis. *S. aureus* MN8 growth was determined by colony counts (CFU/ml).

### 2.6. Post-market safety surveillance

Tampon manufacturers are required in some countries to monitor the safety of marketed products for regulatory compliance reporting (110). Data captured through post-marketing surveillance systems can provide evidence of long-term safety of products. Additionally, post-marketing surveillance data can be used to identify AE changes and new AEs that may occur outside the timeframe of shorter-duration clinical studies.

A post-marketing (passive) surveillance database is maintained to collect, track, and report AEs that consumers, their relatives, or other individuals provide voluntarily. Consumer comments and complaints are collected from various methods including phone calls, e-mail, and company-sponsored Web sites. AEs are ascertained from these consumer comments. An AE is defined by world-wide regulatory agencies as any undesirable effect on an individual's health and/or well-being associated with the use, misuse/overuse (intentional or not), off-label use of a product, or accidental/occupational exposure, whether or not it is considered product related (a causal relationship with the use of the product may not exist). The consumer comments are entered into a central, global database. AE data are coded using the Medical Dictionary for Regulatory Activities (MedDRA) terminology Preferred Terms (PTs). AE data are reviewed by trained individuals and quantified routinely to compare with historic data and identify changes that could suggest a potential safety concern.

We conducted four separate queries of the post-marketing surveillance database, one for each product type assessed in this analysis (TPP, TPC, TC and TCA). The products queried were aligned to the regions where and when specific products were sold. Additionally, the time periods (i.e., years) for each product were limited to no more than 10 years and began when the product appeared in the market for an entire calendar year. The years analyzed were 2015 to 2021 for TPP, 2019 to 2021 for TPC, and 2012 to 2021 for both TC and TCA. We used descriptive statistics to summarize the data. Safety was assessed by analyzing the MedDRA PTs associated with the AEs; multiple PTs may exist for an individual case. We analyzed the most commonly reported AEs and the reporting rates (AE cases per one million tampons shipped; i.e., AE cases normalized to shipments) for four Tampax products separately. The descriptive analysis was exempt from human subjects committee review since it used existing, de-identified data.

## 3. Results of the tampon safety assessment

### 3.1. Biocompatibility

**Figure 3:**
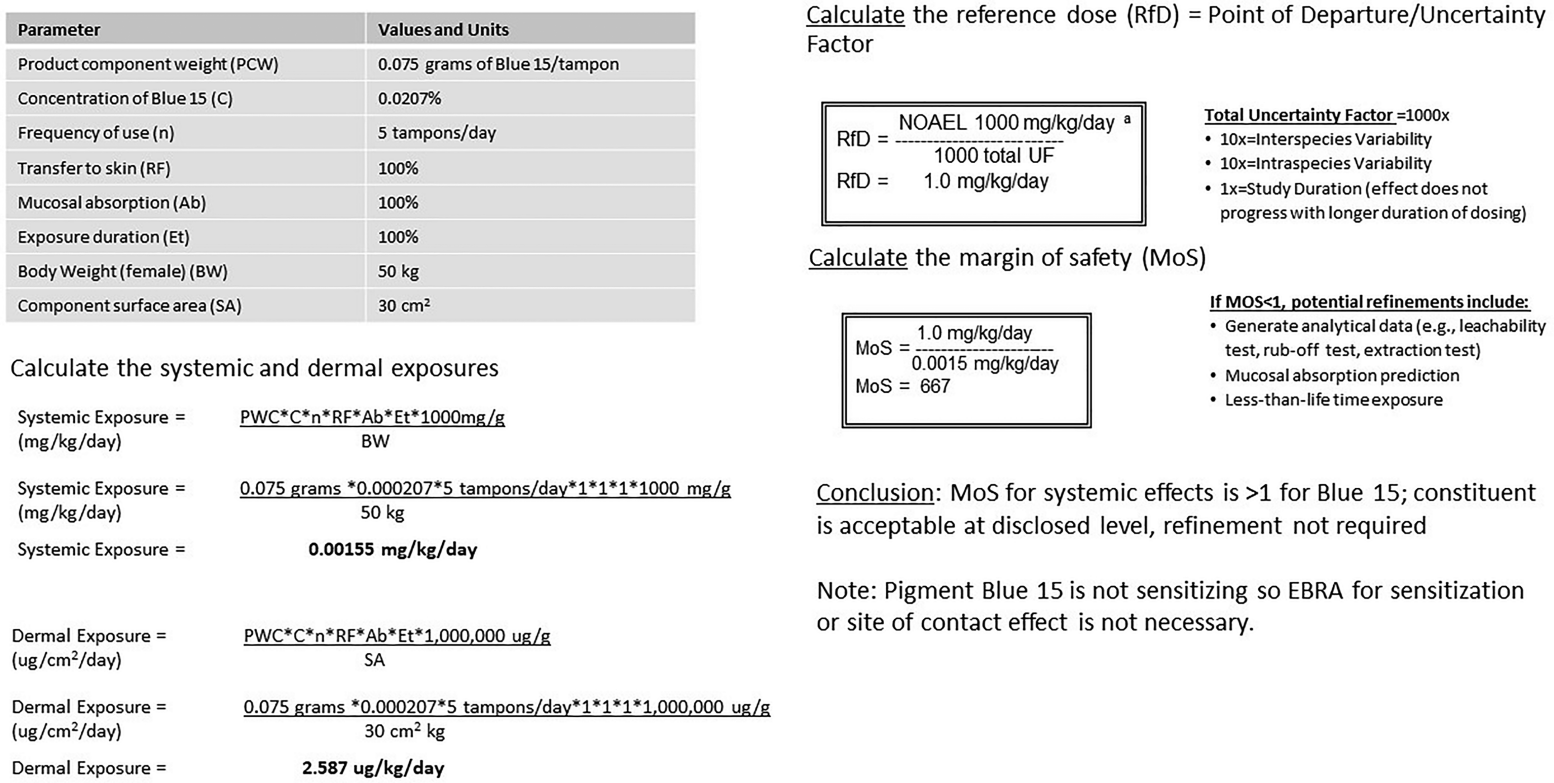
Pigment 15 exposure assessment example.

Smaller molecular weight constituents (processing aid residues, fiber treatment, impurities) identified by raw material supplier disclosure were subject to contemporary quantitative risk assessment as described. All substances had sufficient margins of safety (MOS) to support their presence in final product. An example of one such assessment is found in Figure 3. Blue Pigment 15, (Copper, [29H,31H-phthalocyaninato(2-)-.kappa.N29,.kappa.N30.kappa. N31,.kappa.N32]-, (SP-4-1)-) (Chemical Abstract Service Reference Number: 147-14-8), is a non-volatile solid used as a colorant for surface coatings, printing inks, textile printing, and colored chalks. It is used throughout industry in the coloring of detergents, soaps, and other cleaners as well as of polymers which are intended as coatings on woven, and nonwoven fibers. It is approved for use as a colorant in polypropylene sutures (amount not to exceed 0.5% by weight of the suture) and contact lenses. (21 CFR 74.3045(c)(1)(i); 21 CFR 74.3045 (c)(2). Blue 15 is well characterized (111–115). It is non-genotoxic, as determined by absence of genotoxic response in several *in vitro* bacterial and mammalian mutation as well as cytogenetic assays and in an *in vivo* micronucleus assay. There were no carcinogenic effects in an 8-month subcutaneous carcinogenicity study in mice. OECD (Organisation for Economic Co-operation and Development) cited an estimated dose of low concern of 0.2 mg/kg bw/day based on a NOAEL of 200 mg/kg bw/day in a 28 day repeat dose oral gavage study where effects were limited to a significant decrease of red blood cells at the next highest dose of 1,000 mg/kg bw/day. Subsequent publications first by OECD and then by ECHA suggest these changes were not toxicologically meaningful because they were minor and lacked dose responsiveness. Longer duration (90 day) feeding studies in both rats and mice showed lack of toxicological effects up to an including the highest dose tested (∼>4,500 mg/kg bw/day and ∼16,000 mg/kg bw/day, respectively). In totality, this dataset suggests a low order of repeat dose toxicity for Blue 15; the NOAEL of 1,000 mg/kg bw/day is appropriate as the point of departure (POD). This POD is considered conservative for use in safety assessment given the longer duration studies in the same species which showed higher PODs (116). Blue 15 is not a developmental or reproductive toxicant based on absence of adverse effects on the reproductive ability of parents or any gross malformation in the offspring in a 6-to-8-week oral reproductive screening study in rats receiving doses up to 1,000 mg/kg/day. The undiluted substance is not irritating to the skin, and skin sensitization studies in rats and mice as well as patch testing in humans show Blue 15 is not sensitizing.

A reference dose is calculated by dividing the point of departure (the point on a toxicological dose-response curve established from experimental data or observational data generally corresponding to an estimated low effect level, no adverse effect level or no effect level) by an uncertainty factor that addresses corrections for interspecies variability (10), intraspecies variability (10) and study duration (10). Thus, dividing the NOAEL of 1,000 mg/kg bw/day by 1,000, yields a Pigment Blue 15 RfD of 1.00 mg/kg bw/day. Dividing the RfD by the calculated potential exposure from lifetime tampon usage (0.0015 mg/kg/day) shows a MOS well in excess of 1 (MOS = 667), confirming that this substance can be present at the intended usage level, safely.

### 3.2. Vaginal Mucosa assessment

#### 3.2.1. Visual inspection

The visual inspection of the four test products and their applicators confirmed that pressure points, rough edges and/or sharp contact points were absent. Physical irritation associated with use of these products and designs was unlikely.

#### 3.2.2. Randomized clinical trial of four tampons

Of the 94 subjects randomized to test products, 89 completed the 4-month study treatment phase (4 menstrual period cycles) (Supplementary Figure S1). Five subjects did not complete the treatment phase. One subject was discontinued due to non-compliance with study procedures; the other four were lost to follow-up. The age range was 18–49 (Supplementary Table S1). Most were Caucasian (56%) and Black (39%) with 2% each of Asian Oriental and American Indian/Alaskan Native and 3% other.

Average wear time for each tampon was 4.4–4.6 h with 17.0–17.6 tampons used per period cycle, with no statistically significant differences among wear time for each of the products (Supplementary Table S2).

All AEs reported during study visits (categorized as “possible” or “probable” per the principal investigator, Table 4) were mild in severity and no subjects withdrew due to an AE. Of the possibly and probably-related AEs, itching was the most commonly reported AE (20 occurrences; 10 of these for same subject), followed by cramping (8 occurrences), burning (12 occurrences), and stinging (12 occurrences); other AEs were reported with low incidence or in only one subject. All were self-limited and resolved without treatment. All are considered normal or expected occurrences for menstruating women or tampon users. One serious AE was reported but deemed by the study physician to be not-product related and the subject was able to remain in the study (hospitalization for post-traumatic stress disorder). There were no observations made by the site physician of erythema, abrasions, ulcerations or abnormal vaginal discharge at any post-use evaluations for all tampons (data not shown).

**Table 4:** Treatment-emergent adverse events* randomized subjects.

Category	TPP (*N*^†^ = 91) *n* (%)^‡^ nAE^§^	TPC (*N*^†^ = 92) *n* (%)^‡^ nAE^§^	TC (*N*^†^ = 90) *n* (%)^‡^ nAE^§^	TCA (*N*^†^ = 91) *n* (%)^‡^ nAE^§^
AEs*	9 (9.9%) 9	5 (5.4%) 16	11 (12.2%) 21	13 (14.3%) 35
Serious AEs	1 (1.1%) 1**	0 (0.0%) 0	0 (0.0%) 0	0 (0.0%) 0
Withdrawn Due to AEs	0 (0.0%) 0	0 (0.0%) 0	0 (0.0%) 0	0 (0.0%) 0
**Causality of AE**				
Possibly-Related	0 (0.0%) 0	0 (0.0%) 0	0 (0.0%) 0	2 (2.2%) 2
Probably Related	4 (4.4%) 4	3 (3.3%) 13	5 (5.6%) 14	7 (7.7%) 28

* Only adverse events during product usage were reported.

** Hospitalization for post-traumatic stress disorder: assessed by PI as “Not related to test product”.

^†^ *N*, number of subjects who received products.

^‡^ *n* (%), number and percent of subjects who reported adverse events.

^§^ *n*AE, number of adverse events.

Results from daily diaries showed that all products rated well on comfort: a mean of >70 on a 0–100 scale (Supplementary Table S3). Comfort ratings were statistically significantly better for TPP and TPC as was intended by their design.

Self-reported diary comments of discomfort are described in Supplementary Figure S2. Burning, itching and stinging were infrequent, being reported by only 0% to 4.6% of subjects. Discomfort from insertion, removal and wearing the tampons was reported in 9.4% to 27.2% of subjects with an overall mean range of 2.2% to 13.1% of tampon uses (data not shown). Overall product comfort ranged from 60.9% to 78.4%.

### 3.3. Vaginal Microbiota assessment

#### 3.3.1. *In vitro* assessment of four tampon products on representative vaginal microbiota

All Tampax tampons met the success criteria of <2 log difference of product at both 24 and 48 h compared to the consortium control. Most values were <0.5 log change over the consortium control. The povidone iodine (positive) control perturbed the microflora as expected. The in-market product control (known to not disturb microflora during the test) also demonstrated changes of <2 log difference at all test points for all organisms when compared to the consortium control. Therefore, the test results of the tampon test products are considered valid.

#### 3.3.2. Randomized clinical trial of four tampons: culture-independent microbiome analyses of vaginal fluid

At the baseline assessment, the mean vaginal pH of the 89 subjects completing the randomized clinical trial was 4.22 ± 0.34. The mean pH at the post-use visits ranged from a mean of 4.42–4.50 post-use for all products (Supplementary Table S4). There was a small difference in post-use pH among the products likely attributed to the fact that baseline measurements were taken in between menstrual periods when pH levels are naturally more acidic than during menstruation. All subjects at all study assessment timepoints had vaginal discharge recorded as “normal”.

**Figure 4:**
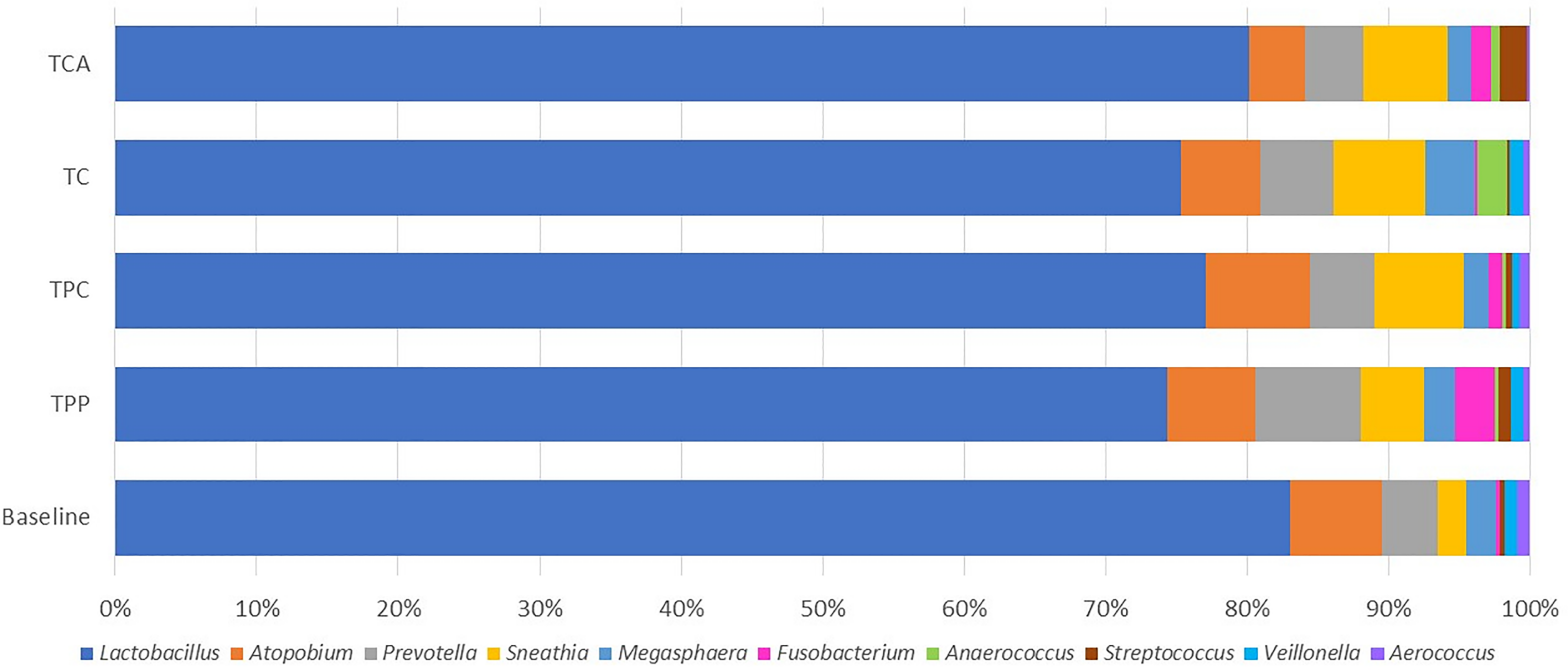
The vaginal microbiota composition at the genus level >0.5%.

Of the 89 subjects who completed the study, 56 had a complete swab sample set for microbiome analysis. The swab set included 5 samples: one taken at baseline (before using any study tampon products) and one after using each of the four tampon products. Figure 4 presents the individual vaginal microbiota genera with average relative abundance >0.5% recorded in the vaginal swabs of women at baseline, i.e., before use of any study tampons, and after the last use of each of the four study tampons (TPP, TPC, TC, or TCA). Lactobacillus was the dominant genus across all samples.

Several of the vaginal bacteria genus showed statistically significant changes in relative abundance through paired Wilcoxon test or paired T test as shown in the Table 5. Meanwhile, only the following differences are significant with False Discovery Rate (FDR) adjusted *p* value less or equal than 0.05: TPP samples have a lower *Lactobacillus* and higher *Streptococcus* abundance than baseline and TC samples have higher *Sneathia* and *Anaerococcus* abundance than baseline. The Kruskal Wallis analyses demonstrated no statistically significant differences based on product use or by study visit day. However, statistically significant differences by study subject were observed for each of the genera assessed. These data indicate that the changes observed in individual genera presence were mainly due to significant woman-to-woman variability and not product use.

**Table 5:** Vaginal microbiota genus composition.

Taxon	Average relative abundance					Kruskal Wallis test *p* value			Paired Wilcoxon test *p* value vs. baseline				Paired *T* test *p* value vs. baseline			
*Genus*	Baseline	TPP	TPC	TC	TCA	Product Use	Visit	Subject	TPP	TPC	TC	TCA	TPP	TPC	TC	TCA
*Lactobacillus*	77.12	68.56	71.04	70.42	75.86	0.50	0.73	**1.65 × 10^−20^**	**0.00**	0.27	0.06	0.62	0.01	0.11	0.03	0.69
*Atopobium*	6.05	5.73	6.83	5.20	3.79	0.85	0.82	**4.98 × 10^−22^**	0.81	0.90	0.44	0.02	0.82	0.67	0.58	0.07
*Prevotella*	3.68	6.83	4.17	4.88	3.84	0.62	0.75	**2.65 × 10^−17^**	0.14	0.35	0.08	0.30	0.07	0.67	0.21	0.90
*Sneathia*	1.86	4.13	5.85	6.02	5.63	0.84	0.66	**2.89 × 10^−23^**	0.06	0.04	**0.01**	0.01	0.15	0.09	0.04	0.02
*Megasphaera*	1.91	2.04	1.57	3.31	1.61	0.95	0.96	**6.3 × 10^−27^**	0.92	0.14	0.04	0.64	0.80	0.48	0.05	0.54
*Fusobacterium*	0.24	2.56	0.89	0.18	1.29	0.80	0.85	**1.19 × 10^−15^**	0.15	0.62	0.37	0.22	0.10	0.22	0.80	0.22
*Anaerococcus*	0.06	0.22	0.25	1.95	0.65	0.16	0.10	**5.19 × 10^−12^**	0.01	0.03	**0.00**	0.01	0.01	0.19	0.08	0.04
*Streptococcus*	0.33	0.80	0.45	0.15	1.80	0.23	0.39	**4.32 × 10^−9^**	**0.01**	0.18	0.08	0.10	0.14	0.81	0.51	0.24
*Veillonella*	0.79	0.84	0.47	0.91	0.01	0.40	0.48	**1.93 × 10^−6^**	0.12	0.70	0.32	0.26	0.83	0.28	0.66	0.22
*Aerococcus*	0.85	0.45	0.66	0.44	0.19	0.66	0.98	**3.9 × 10^−19^**	0.29	0.04	0.19	0.01	0.30	0.55	0.20	0.13

*p* value < = 0.05 in red font, statistically significant values that remain significant after false discovery rate analysis (*p* value < = 0.05) were highlighted with red bold font.

**Figure 5:**
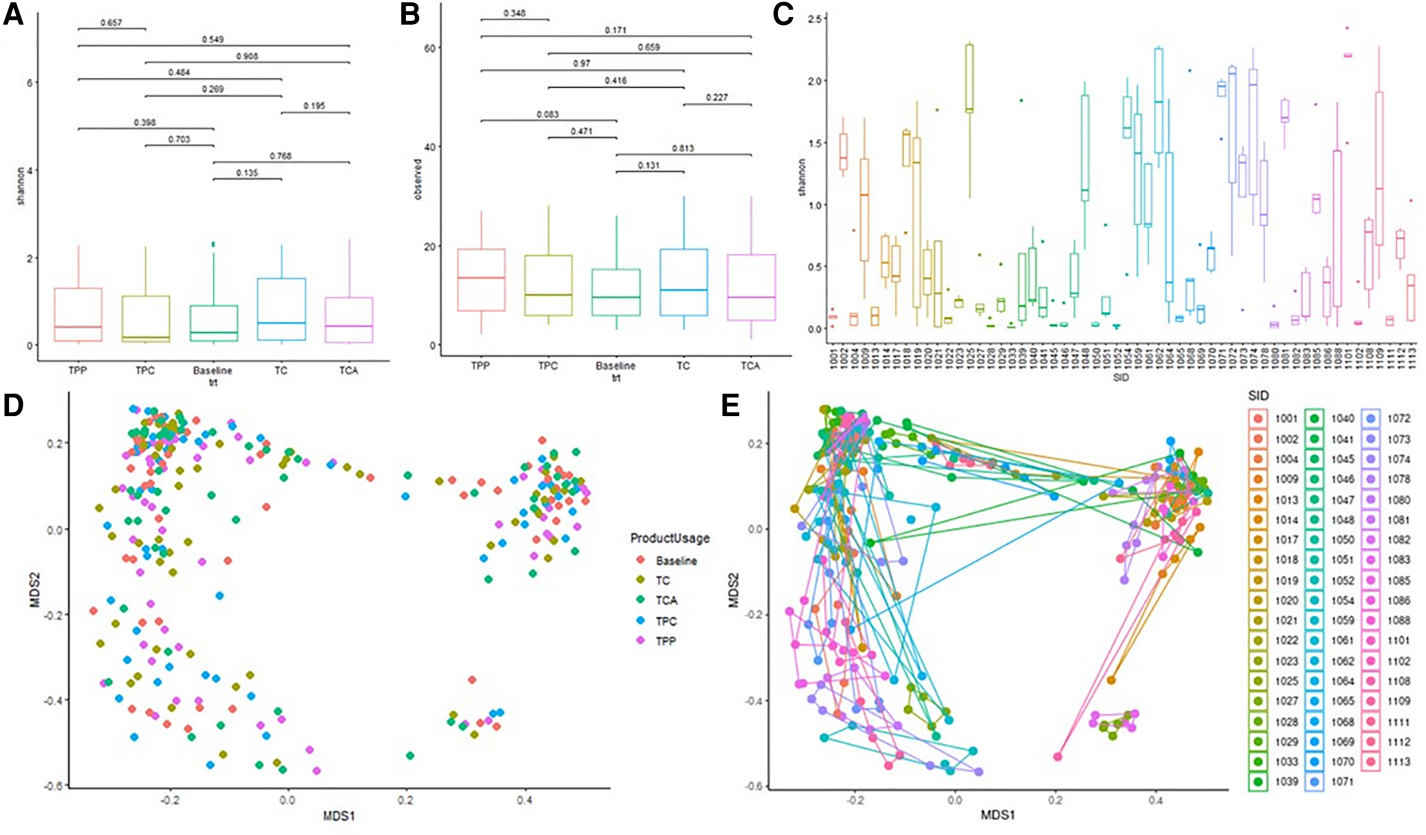
Diversity analysis of the vaginal microbiota. (**A**) Average Shannon diversity measurement by test product. Each colored box represents the Shannon diversity measurement from one sampling point; line within each box represents median. Pairwise student *T*-test *p* values are illustrated above the horizontal line at the top of the graph. (**B**) Average observed Species by test product. Each colored box represents the number of observed species from one sampling point, line within each box represents median. Pairwise student *T*-test *p* values are illustrated above the horizontal line at the top of the graph. (**C**) Average Shannon diversity measurement among different subjects. Each colored box represents the Shannon diversity measurement from one woman's 5 samples with or without tampon usage, line within each box represents median. (**D**) Beta diversity by Bray Curtis Distance: Multi-Dimensional Scaling analysis by product. Each data point represents an entire swab sample compromising many organisms. Each vaginal bacteria community (characterized by 16S rRNA gene sequencing) is plotted against all other communities. (**E**) Beta diversity by Bray Curtis Distance: Multi-Dimensional Scaling analysis by subject. Each subject (*n* = 56) has been assigned an individual color, and for each subject, the 5 swabs are presented. He five swabs for one subject are plotted against all swabs for all other subjects.

Figure 5 shows alpha and beta diversity analyses, by subject or by product use (baseline, and after use of each product). Grouping samples by product usage, there were no significant differences in Shannon (alpha) diversity of microorganism (Figure 5A) (pairwise student *T*-test *p* values all >0.05; Krusalis Wallis *p* = 0.57), nor were there statistically significant differences in alpha diversity measurement of the number of observed genera (Figure 5B) (pairwise t = test p > 0.05, Krusalis Wallis *p* = 0.43). However, there were significant differences in Shannon diversity among the study subjects (Figure 5C) [Pairwise Student T-test results (not shown)] that indicated significant subject-subject differences. Kruskal Wallis *p* = 3.87 × 10^−19^) shows further significant subject-to-subject variability.

MDS analysis (beta diversity), calculated using Bray-Curtis similarities and Adonis testing, compared baseline and post-use samples (Figure 5D). All four product usage samples and baseline were distributed randomly across the plot showing no trend, pattern or statistical significance attributed to tampon use (Adonis test *p* value 0.983). MDS analyses comparing an individual subject's 5 samples verses all other subjects in the study (Figure 5E) clearly shows that for each subject, the respective swabs are clustering together with an Adonis test *p* value of 0.001. Together, Figures 5A–E demonstrate that any vaginal bacteria community diversity differences are likely due to significant variability from subject to subject and not product usage.

**Figure 6:**
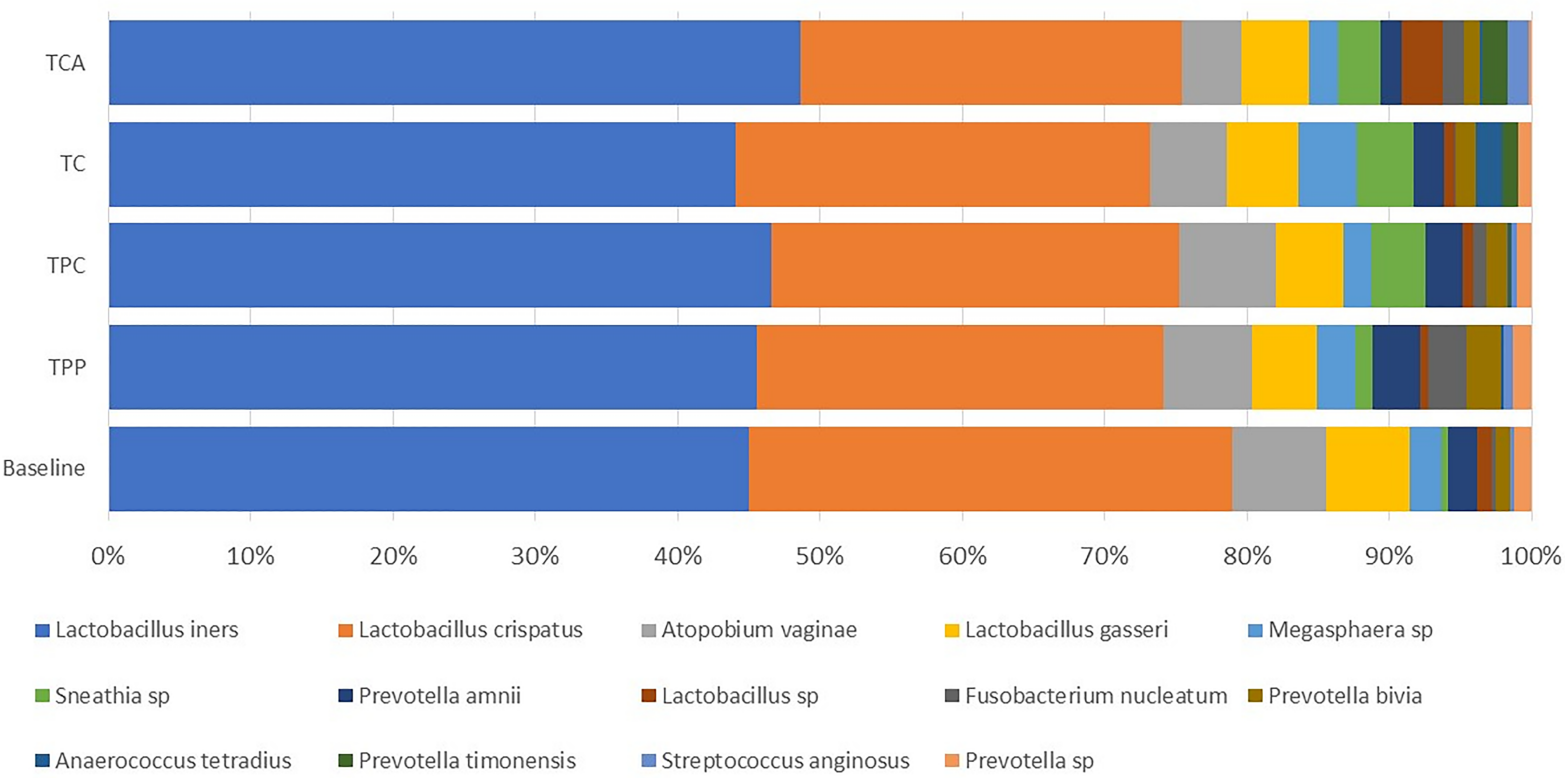
Vaginal Microbiota composition (species level) > 0.5%.

Our final analysis sought to ascertain presence or absence of key species through species analysis (Figure 6, Table 6). *Gardnerella vaginalis, Escherichia coli, Staphylococcus aureus* were not detected in any samples*. Lactobacillus gasseri and Prevotella bivia* were present, but did not show any significant differences in presence attributable to product usage (Krusalis Wallis *p* > 0.05). Instead, there were statistically significant subject-to-subject differences for all species assessed except 1 (*Prevotella timonensis*). Two significant species differences with FDR-adjusted *p* value less or equal than 0.05 were the TC verses baseline and TCA verses baseline comparisons. TC and TCA samples were sequenced in a different batch than the baseline, TPP and TPC samples, thus these differences are likely not true biological differences but rather a batch effect.

**Table 6:** Vaginal microbiota species composition.

Taxon	Average relative abundance					Kruskal Wallis test *p* value		Paired Wilcoxon test *p* value vs. baseline				Paired *T* test *p* value vs. baseline			
Genus	Baseline	TPP	TPC	TC	TCA	Product Use	Subject	TPP	TPC	TC	TCA	TPP	TPC	TC	TCA
*Lactobacillus iners*	40.33	39.65	41.10	39.22	44.70	0.96	**0.00**	0.16	0.77	0.75	0.95	0.86	0.88	0.80	0.23
*Lactobacillus crispatus*	30.45	24.80	25.24	25.97	24.66	0.95	**0.00**	0.21	0.18	0.31	0.39	0.13	0.17	0.24	0.06
*Atopobium vaginae*	5.90	5.42	5.99	4.75	3.90	0.95	**0.00**	0.86	0.46	0.35	0.03	0.76	0.94	0.49	0.13
*Lactobacillus gasseri*	5.30	4.00	4.20	4.44	4.36	0.73	**0.00**	0.32	0.77	0.33	0.13	0.38	0.39	0.35	0.22
*Megasphaera sp*	1.96	2.30	1.74	3.64	1.88	0.92	**0.00**	0.89	0.32	0.02	0.73	0.57	0.63	0.03	0.87
*Sneathia sp*	0.42	1.04	3.32	3.60	2.77	0.76	**0.00**	0.27	0.01	**0.01**	0.02	0.26	0.08	0.08	0.08
*Prevotella amnii*	1.81	2.96	2.30	1.90	1.33	0.71	**0.00**	0.22	0.35	0.12	0.80	0.30	0.43	0.82	0.51
*Lactobacillus sp*	0.91	0.51	0.66	0.68	2.61	0.76	**0.00**	0.42	0.44	0.81	0.12	0.47	0.70	0.66	0.23
*Fusobacterium nucleatum*	0.24	2.33	0.81	0.07	1.41	0.64	**0.00**	0.13	0.94	0.82	0.12	0.14	0.28	0.41	0.23
*Prevotella bivia*	0.92	2.09	1.32	1.23	0.98	0.55	**0.00**	0.16	0.67	0.42	0.99	0.09	0.41	0.61	0.88
*Anaerococcus tetradius*	0.02	0.15	0.18	1.64	0.25	0.25	**0.00**	0.00	0.02	**0.01**	0.04	0.02	0.27	0.13	0.21
*Prevotella timonensis*	0.00	0.01	0.05	1.00	1.62	**0.00**	0.14	1.00	1.00	**0.00**	**0.00**	0.32	0.32	0.01	0.01
*Streptococcus anginosus*	0.29	0.61	0.38	0.05	1.33	0.35	**0.00**	0.01	0.13	0.10	0.04	0.28	0.84	0.38	0.31
*Prevotella sp*	1.08	1.11	0.89	0.82	0.21	0.77	**0.00**	0.59	1.00	0.80	0.05	0.88	0.82	0.61	0.16

*p* value < = 0.05 in red font, statistically significant values that remain significant after false discovery rate analysis (*p* value < = 0.05) red bold font.

### 3.4. *S. aureus* growth and TSST-1 toxin production

The *in vitro* shake flask assay was performed to evaluate the four Tampax study tampons along with a medium control as well as a currently marketed tampon as benchmark control. Growth of *S. aureus* MN8 was statistically significantly reduced vs. the medium control for all test products and the benchmark control (Table 7). Also, all test products and the benchmark control showed significantly lower TSST-1 µg/ml concentrations when compared to the medium control as tested by Western immunoblot analysis (Table 7). Double immunodiffusion data confirmed the Western blot data (data not shown). These data support the conclusion that the Tampax study tampons did not increase *S. aureus* growth or TSST-1 toxin production *in vitro*.

**Table 7:** Effect of tampons on growth of TSS *Staphylococcus aureus* MN8 and TSST-1 production.

Product	Growth of TSS *Staph. aureus* MN8			TSST-1 production (µg/ml)		
	log CFU/ml		*T*-test	Western blots		*T*-test
	Mean	SD		Mean	SD	
Control (medium alone)	10.23	0.08		20.6	4	
In-market benchmark control	10	0.05	0.002	0.5	0.2	0.00001
TPP	9.73	0.12	0.04	0.3	0.1	0.00001
TPC	9.9	0.02	0.00004	1.1	0.6	0.00483
TC	9.73	0.12	0.0001	0.9	0.2	0.00001
TCA	8.93	0.22	0.000004	0.5	0.3	0.00001

CFU, colony forming unit; SD, standard deviation; all tests were performed in replicate (*n* = 5). *T*-Test is compared to the control (medium alone).

### 3.5. Post-market safety surveillance

Table 8 describes the frequency of AE cases associated with the products over a time frame of up to 10 years in-market. In brief, the data indicate very low AE reporting rates with nothing higher than 0.80 AE cases per one million tampons shipped, and all years for TC and TCA at or below 0.14 reported AE cases per one million tampons shipped.

**Table 8:** Frequency and shipment-adjusted reporting rates of reported adverse events associated with four Tampax tampon types, 2012–2021.

Product type	Cases and rates	2012	2013	2014	2015	2016	2017	2018	2019	2020	2021	Total
TPP	Adverse event cases (*N*)	NA*	NA	NA	194	403	249	120	112	82	92	1,252
	Reporting rate**	NA	NA	NA	0.52	0.80	0.44	0.22	0.20	0.18	0.21	0.36
TPC	Adverse event cases (*N*)	NA	NA	NA	NA	NA	NA	NA	24	20	24	68
	Reporting rate	NA	NA	NA	NA	NA	NA	NA	0.68	0.47	0.59	0.57
TC	Adverse event cases (*N*)	50	51	44	73	63	100	50	82	67	54	634
	Reporting rate	0.05	0.05	0.05	0.10	0.08	0.14	0.07	0.11	0.10	0.08	0.08
TCA	Adverse event cases (*N*)	15	26	30	31	41	34	37	16	20	22	272
	Reporting rate	0.02	0.04	0.05	0.06	0.10	0.10	0.12	0.05	0.07	0.08	0.06

* NA, not applicable—product unavailable in market (entire calendar year required) and structured fields unavailable in post-marketing surveillance database.

** Reporting rate = adverse event cases per one million tampons shipped.

A total of 1,252 TPP tampon cases were voluntarily reported between 2015 and 2021 (Table 8). A spike in cases occurred in 2016 (*N* = 403 compared to *N* = 194 in 2015) based on consumer comments indicating that the tampon applicator was not functioning properly during insertion. After the device design was improved, a decline in the number of cases reported occurred through 2021. Shipment-adjusted reporting rates (AE cases per one million tampons shipped) by year were 0.80 or less. Nearly all 1,252 cases were reported in females (*N* = 1,215; 97.0%), with one case reported in males (0.1%) and 36 cases with gender unspecified (2.9%) (Supplementary Table S5). Age group was known for only approximately one-fourth (24.1%) of all cases. Almost all cases were reported from the regions of North America (*N* = 696; 55.6%) and Europe, India, the Middle East, and Africa (EIMEA, *N* = 533, 42.6%). A slightly higher reporting of cases occurred during the spring and summer months in Europe and North America (i.e., second and third quarters). E-mail (*N* = 878; 70.1%) was the most common reporting method.

For TPP, the top five most commonly reported AEs using PT terminology were: complication of device insertion (*N* = 634; 50.6%), vulvovaginal pain (*N* = 498; 39.8%), vulvovaginal discomfort (*N* = 360; 28.8%), vulvovaginal injury (*N* = 298; 23.8%), and foreign body in reproductive tract (*N* = 274; 21.9%) (Table 9). Complication of device insertion events included instances where the applicator collapsed, the user did not insert the tampon high enough into the vagina, or part of the applicator remained in the vagina after tampon insertion. Vulvovaginal injury events included complaints of pinching or cutting by sharp or protruding parts of the plastic applicator. Foreign body in reproductive tract events indicated the consumer was unable to easily remove the tampon from the vagina. The PTs for TPP listed in Table 9 correspond to expected AEs from tampon use. Five cases of menstrual TSS (0.4%) were reported during the seven-year period. Each TPP case included an average of 2.9 AEs (e.g., a consumer reported symptoms of discomfort, pain, and tampon difficult to remove).

**Table 9:** Most frequently reported (top 15) adverse events from post marketing surveillance, by product type, 2012–2021.

	TPP, *N* = 1,252, (2015–2021)			TPC, *N* = 68, (2019–2021)			TC, *N* = 634, (2012–2021)			TCA, *N* = 272, (2012–2021)		
	Preferred term	*n* *	%	Preferred term	*n* *	%	Preferred term	*n* *	%	Preferred term	*n* *	%
1	Complication of device insertion	634	50.6	Vulvovaginal discomfort	23	33.8	Foreign body in reproductive tract	289	45.6	Foreign body in reproductive tract	126	46.3
2	Vulvovaginal pain	498	39.8	Vulvovaginal pain	19	27.9	Vulvovaginal pain	183	28.9	Vulvovaginal pain	88	32.4
3	Vulvovaginal discomfort	360	28.8	Foreign body in reproductive tract	17	25.0	Complication of device removal	151	23.8	Complication of device removal	68	25.0
4	Vulvovaginal injury	298	23.8	Complication of device removal	14	20.6	Vulvovaginal discomfort	141	22.2	Complication of device insertion	54	19.9
5	Foreign body in reproductive tract	274	21.9	Complication of device insertion	11	16.2	Complication of device insertion	133	21.0	Vulvovaginal discomfort	53	19.5
6	Complication of device removal	206	16.5	Vulvovaginal pruritus	8	11.8	Vulvovaginal injury	89	14.0	Vulvovaginal injury	24	8.8
7	Device difficult to use	60	4.8	Hypersensitivity	6	8.8	Device difficult to use	30	4.7	Device use issue	12	4.4
8	Skin discoloration	43	3.4	Vulvovaginal injury	4	5.9	Device use issue	23	3.6	Vulvovaginal pruritus	12	4.4
9	Vulvovaginal burning sensation	30	2.4	Fungal infection	2	2.9	Vulvovaginal burning sensation	23	3.6	Vaginal odor	9	3.3
10	Injury associated with device	29	2.3	Genital discomfort	2	2.9	Vulvovaginal pruritus	21	3.3	Malaise	8	2.9
11	Exposure via skin contact	28	2.2	Pruritus genital	2	2.9	Device use error	11	1.7	Vaginal infection	7	2.6
12	Product package associated injury	26	2.1	Urinary tract infection	2	2.9	Vaginal discharge	11	1.7	Abdominal pain upper	6	2.2
13	Vulvovaginal pruritus	22	1.8	Vaginal disorder	2	2.9	Vaginal odor	11	1.7	Device difficult to use	6	2.2
14	Vaginal odor	19	1.5	Vaginal infection	2	2.9	Toxic shock syndrome	10	1.6	Vulvovaginal burning sensation	6	2.2
15	Vaginal hemorrhage	15	1.2	Vulvovaginal burning sensation	2	2.9	Abdominal pain	9	1.4	Genital pain	5	1.8
				Vulvovaginal inflammation	2	2.9	Abdominal pain upper	9	1.4	Vaginal discharge	5	1.8
				Vulvovaginal rash	2	2.9	Vaginal hemorrhage	9	1.4			
							Vaginal infection	9	1.4			

* Each case may report one or more adverse events.

A total of 68 TPC tampon cases were voluntarily reported between 2019 and 2021 (Table 8). The number of cases reported remained steady from 2019 to 2021 (range 20 to 24). Shipment-adjusted reporting rates (AE cases per one million tampons shipped) by year were 0.68 or less. Nearly all 68 cases were reported in females (*N* = 66; 97.1%), with zero cases reported in males and two with gender unspecified (2.9%) (Supplementary Table S5). Age group was known for less than one-fifth (19.1%) of all cases. Over half of all cases were reported in North America (*N* = 39; 57.4%). Most of the cases were reported during the first three quarters of the year (i.e., January through September). E-mail (*N* = 31; 45.6%), phone (*N* = 12; 17.6%), and company-sponsored Web sites (*N* = 12; 17.6%) were the preferred reporting methods.

For TPC, the top five most commonly reported AEs were: vulvovaginal discomfort (*N* = 23; 33.8%), vulvovaginal pain (*N* = 19; 27.9%), foreign body in reproductive tract (*N* = 17; 25.0%), complication of device removal (*N* = 14; 20.6%), and complication of device insertion (*N* = 11; 16.2%) (Table 9). TPC tampons have an organic cotton core and are often used by consumers who are concerned with hypersensitivity. AEs of hypersensitivity (*N* = 6, 8.8%; Table 9) indicated reports of allergic reactions, which frequently are not medically confirmed. Consumers buying TPC tampons may have heightened perceptions of possible AEs, yet the yearly reporting rates were low (Table 8). The PTs for TPC listed in Table 9 correspond to expected AEs from tampon use. Zero cases of menstrual TSS were reported during the three-year period. Each TPC case included an average of 2.8 AEs.

A total of 634 TC tampon cases were voluntarily reported between 2012 and 2021 (Table 8). The number of reported cases annually ranged from 44 to 100 during the 10-year period. Shipment-adjusted reporting rates (AE cases per one million tampons shipped) by year were 0.14 or less. The reported case count rose to 100 in 2017, and the shipment-adjusted rate was the highest (0.14 AE cases per one million tampons shipped). Nearly all 634 cases were reported in females (*N* = 625, 98.6%), with one case reported in a male (0.2%) and eight cases with gender unspecified (1.3%) (Supplementary Table S5). Age group was known in only 39.3% of all cases. Over 80% of the cases reported came from EIMEA (*N* = 535, 84.4%). A slightly higher reporting of cases occurred from July to September (*N* = 183, 28.9%). E-mail (*N* = 406, 64.0%) and phone (*N* = 173, 27.3%) were the preferred reporting methods.

For TC, the top five most commonly reported AEs were: foreign body in reproductive tract (*N* = 289; 45.6%), vulvovaginal pain (*N* = 183; 28.9%), complication of device removal (*N* = 151; 23.8%), vulvovaginal discomfort (*N* = 141; 22.2%), and complication of device insertion (*N* = 133; 21.0%) (Table 9). Complication of device removal events indicated problems with the cord (e.g., too short, could not find, broke off), complaints that the tampon expanded too much, or that pieces of the tampon broke off in the vagina or during removal. The PTs for TC listed in Table 9 correspond to expected AEs for tampon use. Ten cases of menstrual TSS (1.6%) were reported during the 10-year period. Each TC case included an average of 2.6 AEs.

A total of 272 TCA tampon cases were voluntarily reported between 2012 and 2021 (Table 8). Case counts were consistent across the 10-year period, with the fewest number reported in 2012 (*N* = 15) and greatest reported in 2016 (*N* = 41). Shipment-adjusted reporting rates (AE cases per one million tampons shipped) by year were 0.12 or less. Nearly all 272 cases were reported in females (*N* = 266, 97.8%), with two cases reported in males (0.7%) and four cases with gender unspecified (1.5%) (Supplementary Table S5). Age group was known in only 38.2% of all cases. Almost three-fourths of the cases reported came from EIMEA (*N* = 200, 73.5%). The cases were fairly evenly distributed across reporting quarter. E-mail (*N* = 158, 58.1%) and phone (*N* = 99, 36.4%) were the preferred reporting methods.

For TCA, the top five most commonly reported AEs were: foreign body in reproductive tract (*N* = 126; 46.3%), vulvovaginal pain (*N* = 88; 32.4%), complication of device removal (*N* = 68; 25.0%), complication of device insertion (*N* = 54; 19.9%), and vulvovaginal discomfort (*N* = 53; 19.5%) (Table 9). The PTs for TCA listed in Table 9 correspond to expected AEs for tampon use. Four cases of menstrual TSS (1.5%) were reported for TCA during the ten-year period. Each TCA case included an average of 2.6 AEs.

## 4. Discussion and conclusion

We describe a comprehensive safety assessment approach that assures tampons can be used safely. The approach is illustrated through the example of assessing the safety of four Tampax tampon products. The approach incorporates four segments that comprise the essential subcategories for tampon premarket safety assessments which include: biocompatibility and chemical safety of the product components; physical effects to the vaginal mucosa; effects on the vaginal microbiota; and risk of TSS. Post marketing surveillance provides further evidence of long-term safety of products and can confirm the outcome of the safety assessment.

Elements of safety assessment have been briefly described previously for tampons (10, 11). Similar approaches have been applied to other consumer products (45, 61, 117–119). This report is the first to describe a comprehensive approach and the scientific basis for each element assuring the safe use of tampons as well as offering specific examples of how assessments within each element may be executed. It is important to underscore the word “examples” because different state of the art methods could be used instead. This is particularly important as the state of the science evolves. For example, early Tampax clinical studies used culture-dependent methods when that work was considered the then-state of the art (10, 11). Today, culture-independent methods are being applied.

### 4.1. Biocompatibility/chemical safety assessment

“What's in this tampon” is neither a new question nor unanswered. It is the foundation for a rigorous safety assessment. Detailed information beyond “cotton” or “rayon” (for example, raw material processing aids, purification processes, possible impurities) deep within the raw material supply chain informs the basis of this knowledge. Raw material supplier's confidential disclosures provide these data.

Compositional data inform the biocompatibility/chemical safety assessment. Recognized biocompatibility assessments addressing all toxicological endpoints assures materials that come in contact with the body are non-irritating and non-sensitizing, and if able to be absorbed into the body are supported by sufficient margins of safety (>1). Only with this documentation are these materials deemed safe for use in tampons at the levels present.

Close partnership with manufacturing drives awareness and assessment of potential contaminants. Finished product manufacturing may introduce trace substances (e.g., process aids, residual monomers) that are included within the biocompatibility/chemical safety assessment. If present, these substances must also have a MOS > 1. Tight controls over product manufacturing and strict compliance with good quality manufacturing principles prevent undocumented and unassessed changes to finished product composition. No manufacturing process changes can occur without a reassessment of the impact on the biocompatibility and chemical safety assessments of the product.

The composition of the four product tampons assessed in this paper shows the vast majority of substances present in the finished product (99.45%) are large molecular weight polymers, unable to pass the vaginal mucosa (cotton, rayon, polyethylene/polypropylene, polyester). These large molecular weight materials are non-irritating and non-sensitizing. The biocompatibility/chemical safety assessment of the remaining small molecular weight substances yields a MOS exceeding 1, (as exemplified by the assessment of Pigment blue 15) which supports daily exposure for a menstrual lifetime. Thus, the scientific support for trace raw materials that meet this toxicological limit supports their safe presence in tampons.

### 4.2. Vaginal Mucosa assessment

Reports of vaginal mucosal tissue effects from tampon usage or applicator insertion remain limited (63, 120–122). Vaginal mucosal effects reported in the literature were benign and transient, or attributed to misuse. Subjective product comfort assessments confirm few reports of discomfort attributed to product use (10, 11, 64). The paucity of documented effects aside, new or modified tampons or tampon applicators are assessed for the potential to alter the likelihood of vaginal effects such as abrasions, lesions, or other physical effects or an unacceptable subjective sensory assessment after using the product.

Simple tactile assessment of the product and applicator (for example, rough edges or pinching risk) addresses gross tactical issues that can be rectified prior to any in-use experience. Tactile concerns (abrasions, lesions, ulcerations, etc.) are assessed by visual examination of the tissues after insertion and wear of the product. User experience captured in usage diaries enables researchers to further assess subjective measures of user's comfort with the product during use. Comfort can reflect the user's experience vs. their usual product and while preferences for one product may be greater than others, these data inform more on the consumer preference rather than an indication of their perceived safety.

The manual assessment of the tampon and visual assessment of the vaginal tissue after use confirm the tolerability of the four tampon products assessed in this report. All products lacked physical attributes that could contribute to vaginal lesions or contusions. All products were devoid of any association with erythema, ulcerations or abrasions. Experience and sensorial reports captured by diary further corroborate the tolerability of the tested tampons. There were few reports of burning, stinging, or itching and overall comfort ratings were favorable for all test products. Adverse events were few, mild, and resolved without treatment. Thus, all four study tampons, based on the objective and subject assessment, could be considered to have no adverse effects on the vaginal mucosa and considered comfortable to use.

Prospective clinical trials are challenged by their limited duration (often just 1 menstrual cycle per product). Given that the four study tampons were of similar (but not identical) composition, the prospective clinical trial reported in this study was effectively a four-month assessment of products in-use and thus further lends support to the conclusion that products of this type are quite tolerable with limited to no apparent adverse effects to the vaginal mucosa.

### 4.3. Vaginal microbiota

Assuring new tampon products do not adversely affect the vaginal microbiome is essential to assure the products will not alter the natural composition of this complex community. Most published studies exploring tampon effects on the vaginal microbiome used the then-state-of-the-art clinical microbiology laboratory methods (10, 123, 124). These studies confirmed that tampons could be used safely and without adverse effect on the vaginal microbiota.

Since the advent of culture-independent methodologies, research has largely focused on advancing the understanding of the healthy vaginal microbiome. Of the five identified distinct different vaginal communities, four are dominated by varying lactobacillus species; the fifth group—a diverse community with respect to the first four groups with no clear dominant organism present—still had a Lactobacillus species present in up to 78% of the communities (73). Age, menarche, menses, pregnancy, sexual activity, and douching (125–130) are among the many factors that may alter the structure and composition of the vaginal ecosystem.

*In vitro* methods provide an initial screen for potential effects on vaginal microbiota. The method described in this paper was initially developed in collaboration between P&G and Microbiology Specialists Incorporated (131). The organisms assessed represent the dominant organism of the healthy vagina and several others linked to gynecological diseases or infection including bacterial vaginosis, pelvic inflammatory disease, urogenital infection, vaginal yeast infection, and Toxic Shock Syndrome.

The four tampons assessed in this study had no unfavorable effects on the vaginal organisms, *in vitro.* Each product met the success criteria of <2 log and most were within 0.5 log change over that of the control. These data mirrored the benchmark control, providing confidence that these products would not adversely alter these organisms when used.

Only one other paper has published similar data assessing the impact of a vaginally inserted product on vaginal microorganisms, *in vitro* (61). Expanding this dataset with further assessment of tampons and broadening its application to a variety of other gynecological products will go far to build our understanding of broad utility of this method.

Assessment of vaginal pH and vaginal discharge are broad measures of a healthy vaginal microbiota. Normal vaginal pH is typically 4.0–4.5 (mid cycle) and becomes less acidic during menses. Normal vaginal discharge is described as white or transparent, thick or thin, and mostly odorless (132). The results of our study were consistent with normal pH and discharge assessment, providing initial confidence of no perturbations to the vaginal microbiome.

Assessing effects of tampons on the vaginal microbiome using culture-independent methods is in its infancy and there remains much to be learned about conducting these studies. To date, only two clinical studies have been reported that deployed culture-independent methods assessing the impact of tampons on the vaginal microbiome. Both studies were longitudinal assessments over several menstrual cycles, however only one -Hickey (67)- included a cycle of exclusive pad usage with which to make a meaningful comparison or judgement of potential effects associated with tampon usage. This study showed a shift in the vaginal microbiota composition during menses unrelated to tampon use. The magnitude of that shift was both variable across subjects and transient. Tampon use did not impact the vaginal microbiota.

As expected, in our study Lactobacillus genera were the dominant organisms present across all subjects and all samples, consistent with prior reports of the normal, healthy vaginal biome (73). The other dominant organisms observed were consistent with other work (61). *S. aureus* was undetected in all samples. There were some statistically significant differences in genus or individual species composition between baseline and after product use samples that were attributed to subject-to-subject variability. Similarly, alpha and beta diversity measures also identified significant subject-to-subject diversity across the study population. Importantly, there was no evidence suggesting any of the study tampons assessed affected the microbiome organisms present or the diversity of the community in any of the study subjects.

The assessment of tampons and the vaginal microbiome warrants further study. Future studies would benefit from obtaining baseline and post-product use samples on the same day of the menstrual cycle to ascertain the impact of the menstrual cycle on the microbiome composition. Limiting study population variability (race, age, smoking status, for example) would further limit potentially confounding factors and may reduce some of the subject-to-subject variability observed in our study. Similarly, tighter restrictions on subject habits that are known to lead to loss of Lactobacillus species such as a vegetarian diet or intense exercise (78) will expand our understanding of the microbiome as well. Lastly, studies designed specifically to address microbiome resiliency to the natural shifts that occur with menses will expand our understanding of a normal, possibly hormone-related ebb and flow of organisms present.

### 4.4. Toxin testing

The association of increased risk of menstrual TSS with tampon usage in the 1980s established the need to address TSS risk when evaluating the safety of new tampon products (15, 16). Virtually all cases of tampon-associated menstrual TSS are caused by strains of *S. aureus* that produce the TSST-1 toxin in women who lack protective antibodies. Tampons neither introduce *S. aureus* (133–135), nor are they causative, as TSS occurs menstrually without use of tampons as well as among men, boys, and non-menstruating women (14, 20, 26, 27). Several *in vitro* methods have been developed to address a new or modified tampon's potential to affect *S. aureus* growth and TSST-1 toxin production, *in vitro*. No single test is considered the “gold standard” for toxin testing. Methods should address a new product or material's propensity to enhance the growth of *S. aureus* and increase the production of TSST-1, *in vitro* (28). Toxin testing using the method developed by Schlievert (16, 60) confirms that the four products assessed in this report had reduced *S. aureus* growth, and TSST-1 toxin production vs. medium alone, and performed similarly to an in-market product with no known increased risk of TSS. Thus, these data provide confidence the tampons and the materials they are composed of, should not affect the risk of TSS differently than other tampons in the market.

TSS is a rare illness and thus it is difficult to fully manage the risk of TSS from *in vitro* testing, alone. Awareness of increased risk of TSS associated with tampon use, potential signs and symptoms of the illness, and ways to reduce risk of TSS are essential to helping assure the safe use of tampons. This information is provided with all Tampax tampons sold around the world, even in countries where this information is not required. This information is also available on Tampax websites.

### 4.5. Post-Marketing safety surveillance

Another important element of a product's safety assessment includes data obtained once the product is in-market. These post-marketing surveillance data obtained from voluntary reports provide evidence that the safety assessment process enabled a satisfactory conclusion that the products can be used safely. Most commonly reported complaints included vulvovaginal pain and discomfort, complication of device insertion or removal, and foreign body in reproductive tract. The reported AEs were consistent with the published literature, clinical/consumer learnings as well as the health effects that would be expected based on a tampon being an intravaginal device (e.g., vulvovaginal pain, discomfort, burning sensation). Moreover, the number of menstrual TSS cases reported across all products (*N* = 19) was low.

This analysis provides an AE profile for each of the four brands (TPP, TPC, TC, and TCA) generated from a manufacturer's post-marketing surveillance system. Post-marketing surveillance is not designed to compare data across products. With data provided voluntarily by consumers, the process of generating the data does not allow for analytical comparisons. We therefore analyzed the reported data for each product separately.

External sources also collect post-marketing data. For example, the U.S. Food & Drug Administration's Manufacturer and User Facility Device Experience (MAUDE) database collects medical device reports by mandatory (e.g., manufacturers) and voluntary (e.g., healthcare professionals, consumers) reporting. MAUDE along with post-marketing clinical studies are reviewed for Tampax tampons, and the results augmented to the findings here. A complete AE profile benefits from having post-marketing data from multiple sources.

One reason for conducting post-marketing surveillance is to indicate whether product changes are needed once the product is in market. During the early years of marketing TPP, an increase in consumer comments (*N* = 403 in 2016 vs. *N* = 194 cases in 2015) indicated that the TPP tampon applicator was not functioning properly during insertion into the vagina (PT = complication of device insertion). Post-marketing surveillance helped identify the issue and a redesign of the applicator to mitigate the problem was initiated. Moreover, skin discoloration complaints for TPP (*N* = 43, 3.4%) and TC (*N* = 7, 1.1%; data not shown) revealed that the ink on the tampon wrapper was transferring to the consumers' fingers. While it was determined that there was no objective safety concern associated with the ink transfer, the voluntary information provided by consumers resulted in changing the ink used on wrappers. These examples illustrate the importance of collecting post-marketing surveillance data to identify potential product concerns and to implement product changes and improvements.

Our analysis has several limitations. As with any use of passive surveillance data (i.e., voluntary reports), an under-reporting of AE cases may have occurred. Without actively following tampon users, the true total number of users (i.e., the target population) is unknown. This “denominator problem” prohibits calculating incidence or prevalence. We therefore are limited in calculating statistical analyses and estimated reporting rates using shipment data as a proxy for tampon use (i.e., AE cases per one million shipped tampons). This normalization using shipment data provides context around the variation in the number of AE cases reported each year within a Tampax product type. Additionally, surveillance data may be miscategorized and result in misclassification. For instance, a Tampax product may have been reported or coded incorrectly based on limited information provided by the consumer. Moreover, the voluntary data may be incomplete. In this review, more complete information appears for gender/anatomy than age as the reporter may use words that describe female gender, including “my daughter”, “used in my vagina”, or “used for my period”. Furthermore, the AE reports are unverified by medical professionals. A feature of TPP tampons is the chevron (“V” shape at end opposite of withdrawal cord) design that consumers sometimes mistake for the tampon being broken when they remove it from the vagina. As a result, they fear that pieces have been retained in the vagina and thus the AE would be captured in the MedDRA PT as “Foreign body in reproductive tract”. The data are also prone to biases caused by external influences (e.g., product promotions, litigation, social media posts), product reimbursement requests (e.g., reporting an AE to seek product refund), and consumer awareness (e.g., perceptions of consumers purchasing TPC tampons may trigger them to report specific AEs such as hypersensitivity).

Interpreting the results from post-marketing (passive) surveillance data requires caution. Increases in AEs over time may not represent true increases since, for example, product promotions can increase the number of cases reported. Therefore, in addition to these limitations, business product knowledge should be included when assessing safety. Number of tampons shipped, tampon usage, and reporting of AEs can vary regionally. Moreover, product marketing life within regions impacts reporting. For instance, TC tampons were discontinued in North America when TPP tampons were launched in 2015. We provide a descriptive overview to assess AEs voluntarily reported on four Tampax tampon products throughout the world.

Post-marketing surveillance data are often underutilized. While manufacturing companies may focus efforts on using surveillance data for completing regulatory submissions and compliance, the data can also be useful for further product understanding. Here we show that post-marketing surveillance data can be used to develop an adverse event profile for Tampax tampons which is important since this type of safety information does not currently exist in the literature.

## 5. Overall conclusion

A comprehensive, science-based safety assessment approach is described and illustrated through the example of assessing four products. This approach has assured authorities through regulatory submissions (e.g., 510(k) clearance) that tampons assessed using this approach, can be used safely. This four-part safety assessment approach includes biocompatibility and chemical safety of the product components; physical impacts to the vaginal mucosa; impact to vaginal microbiota; and risk for Toxic Shock Syndrome (TSS). Post-marketing surveillance data enable discovery of product concerns, as well as evidence of the safety assessment that products can be used safely.

## Data Availability

The microbiome (16S) data has been made publicly available under number ‘PRJNA955708’. Data will be released on 2024-04-12.
