# Supplementary material for: The safety assessment of tampons: illustration of a comprehensive approach for four different products
Source: Front Reprod Health. 2023 Jun 20;5:1167868. doi: 10.3389/frph.2023.1167868 (PMC10319135; doi:10.3389/frph.2023.1167868)
Supplement: Supplementary file 1 [file Datasheet1.docx]

**Supplementary Table S1: Demographics (Randomized Subjects)**

| **Measures** | **Result** |
| --- | --- |
| **Ethnicity** | |
| Hispanic/Latino | 2 (2.1%) |
| Not Hispanic or Latino | 92 (97.9%) |
| **Race** | |
| American Indian or Alaskan Native | 2 (2.1%) |
| Asian Oriental | 2 (2.1%) |
| Black | 36 (38.3%) |
| Caucasian | 51 (54.3%) |
| Other | 3 (3.2%) |
| **Age (Years)** | |
| Number of Subjects | 94 |
| Mean | 33.4 |
| Median | 33.0 |
| Min - Max | 18.0 - 49.0 |
| **Body Mass Index** | |
| Number of Subjects | 94 |
| Mean | 31.7 |
| Median | 31.0 |
| Min - Max | 17.0 - 72.0 |

**Supplementary Table S2 Tampon Wear Time and Number Used**

|  | | | | **2-sided P-value** | | |
| --- | --- | --- | --- | --- | --- | --- |
|  | **Number of Subjects** | **Mean** | **Standard Error** | **TPC** | **TC** | **TCA** |
| **Wear Time** | | | | | | |
| TPP | 89 | 4.5 | 0.15 | 0.4049 | 0.3482 | 0.7063 |
| TPC | 89 | 4.4 | 0.15 |  | 0.0781 | 0.2269 |
| TC | 89 | 4.6 | 0.15 |  |  | 0.5739 |
| TCA | 90 | 4.5 | 0.15 |  |  |  |
| **Number of Uses** | | | | | | |
| TPP | 89 | 17.6 | 0.55 | 0.6395 | 0.2142 | 0.1901 |
| TPC | 89 | 17.4 | 0.55 |  | 0.4408 | 0.4004 |
| TC | 89 | 17.0 | 0.55 |  |  | 0.9452 |
| TCA | 90 | 17.0 | 0.54 |  |  |  |
| Wear time (hours) was analyzed with a linear mixed model with effects for treatment, visit, and subject (random). | | | | | | |

**Supplementary Table S3: Comparison of Overall Comfort (Randomized Subjects)**

|  | | | | **Product Comparison P-values^*^** | | |
| --- | --- | --- | --- | --- | --- | --- |
| **Products** | **Number of Subjects** | **Mean^*^** | **Standard Error** | **TPC** | **TC** | **TCA** |
| TPP | 89 | 82.0 | 2.54 | 0.2116 | **0.0027** | **0.0011** |
| TPC | 89 | 78.2 | 2.54 |  | 0.0770 | **0.0409** |
| TC | 89 | 72.7 | 2.54 |  |  | 0.7840 |
| TCA | 90 | 71.8 | 2.52 |  |  |  |
| ^*^Comfort ratings were analyzed with a linear mixed model with effects for treatment, visit, and subject (random). | | | | | | |

**Supplementary Table S4   Vaginal pH Statistics (Randomized Subjects)**

| **Product** | **Number of Subjects^*^** | **Mean** | **Standard Error** | **Median** | **Min** | **Max** |
| --- | --- | --- | --- | --- | --- | --- |
| **Baseline pH** | | | | | | |
| Baseline pH | 89 | 4.22 | 0.034 | 4 | 3.5 | 5 |
| **Post-treatment pH (within 72 hours last tampon use)** | | | | | | |
| TPP | 89 | 4.43 | 0.052 | 4.5 | 4 | 6 |
| TPC | 91 | 4.50 | 0.076 | 4.5 | 4 | 8.5 |
| TC | 87 | 4.48 | 0.067 | 4.5 | 4 | 8 |
| TCA | 86 | 4.42 | 0.059 | 4.25 | 4 | 6.5 |
| ^*^ Six subjects' vaginal pH were not collected due to the menstrual bleeding. | | | | | | |

**Supplementary Table S5. Characteristics of reported adverse event cases associated with four Tampax tampon products, by product type , 2012-2021**

| **Characteristic** | **TPP**  **N=1,252**  **(2015-2021)** | | **TPC**  **N=68**  **(2019-2021)** | | **TC**  **N=634**  **(2012-2021)** | | **TCA**  **N=272**  **(2012-2021)** | |
| --- | --- | --- | --- | --- | --- | --- | --- | --- |
|  | **n** | **%** | **n** | **%** | **n** | **%** | **n** | **%** |
| Gender |  |  |  |  |  |  |  |  |
| Female | 1215 | 97.0 | 66 | 97.1 | 625 | 98.6 | 266 | 97.8 |
| Male | 1 | 0.1 | 0 | 0 | 1 | 0.2 | 2 | 0.7 |
| Unknown | 36 | 2.9 | 2 | 2.9 | 8 | 1.3 | 4 | 1.5 |
|  |  |  |  |  |  |  |  |  |
| Age group |  |  |  |  |  |  |  |  |
| Adolescent (12 to 17 years) | 27 | 2.2 | 1 | 1.5 | 29 | 4.6 | 6 | 2.2 |
| Adult (18 to 64 years) | 273 | 21.8 | 12 | 17.6 | 219 | 34.5 | 98 | 36.0 |
| Elderly (65 years +) | 1 | 0.1 | 0 | 0 | 1 | 0.2 | 0 | 0 |
| Unknown | 951 | 76.0 | 55 | 80.9 | 385 | 60.7 | 168 | 61.8 |
|  |  |  |  |  |  |  |  |  |
| Region |  |  |  |  |  |  |  |  |
| Asia | 22 | 1.8 | 17 | 25.0 | 2 | 0.3 | 36 | 13.2 |
| EIMEA* | 533 | 42.6 | 12 | 17.6 | 535 | 84.4 | 200 | 73.5 |
| Latin America | 1 | 0.1 | NA | NA | 66 | 10.4 | 36 | 13.2 |
| North America | 696 | 55.6 | 39 | 57.4 | 31 | 4.9 | NA | NA |
|  |  |  |  |  |  |  |  |  |
| Quarter |  |  |  |  |  |  |  |  |
| First (January – March) | 267 | 21.3 | 20 | 29.4 | 166 | 26.2 | 65 | 23.9 |
| Second (April – June) | 314 | 25.1 | 25 | 36.8 | 161 | 25.4 | 65 | 23.9 |
| Third (July -September) | 373 | 29.8 | 16 | 23.5 | 183 | 28.9 | 76 | 27.9 |
| Fourth (October – December) | 298 | 23.8 | 7 | 10.3 | 124 | 19.6 | 66 | 24.3 |
|  |  |  |  |  |  |  |  |  |
| Primary Reporting Source** |  |  |  |  |  |  |  |  |
| E-mail | 878 | 70.1 | 31 | 45.6 | 406 | 64.0 | 158 | 58.1 |
| Letter | 11 | 0.9 | 0 | 0 | 18 | 2.8 | 5 | 1.8 |
| Phone | 212 | 16.9 | 12 | 17.6 | 173 | 27.3 | 99 | 36.4 |
| Reviews | 21 | 1.7 | 4 | 5.9 | 0 | 0 | 1 | 0.4 |
| Social Media (company-sponsored) | 110 | 8.8 | 8 | 11.8 | 23 | 3.6 | 6 | 2.2 |
| Via Mailed Survey | 1 | 0.1 | 0 | 0 | 0 | 0 | 0 | 0 |
| Web Site (company-sponsored) | 14 | 1.1 | 12 | 17.6 | 0 | 0 | 0 | 0 |
| Unknown | 5 | 0.4 | 1 | 1.5 | 14 | 2.2 | 3 | 1.1 |

NA = Not applicable, product not sold in region.

*EIMEA = Europe, India, the Middle East, and Africa.

**The primary reporting source was provided when multiple sources were reported. Reviews = Report from ratings/reviews site.

Note. Percentages may not add to 100.0 due to rounding.
