# Supplementary figures and images for: The safety assessment of tampons: illustration of a comprehensive approach for four different products
Source: Front Reprod Health. 2023 Jun 20;5:1167868. doi: 10.3389/frph.2023.1167868 (PMC10319135; doi:10.3389/frph.2023.1167868)

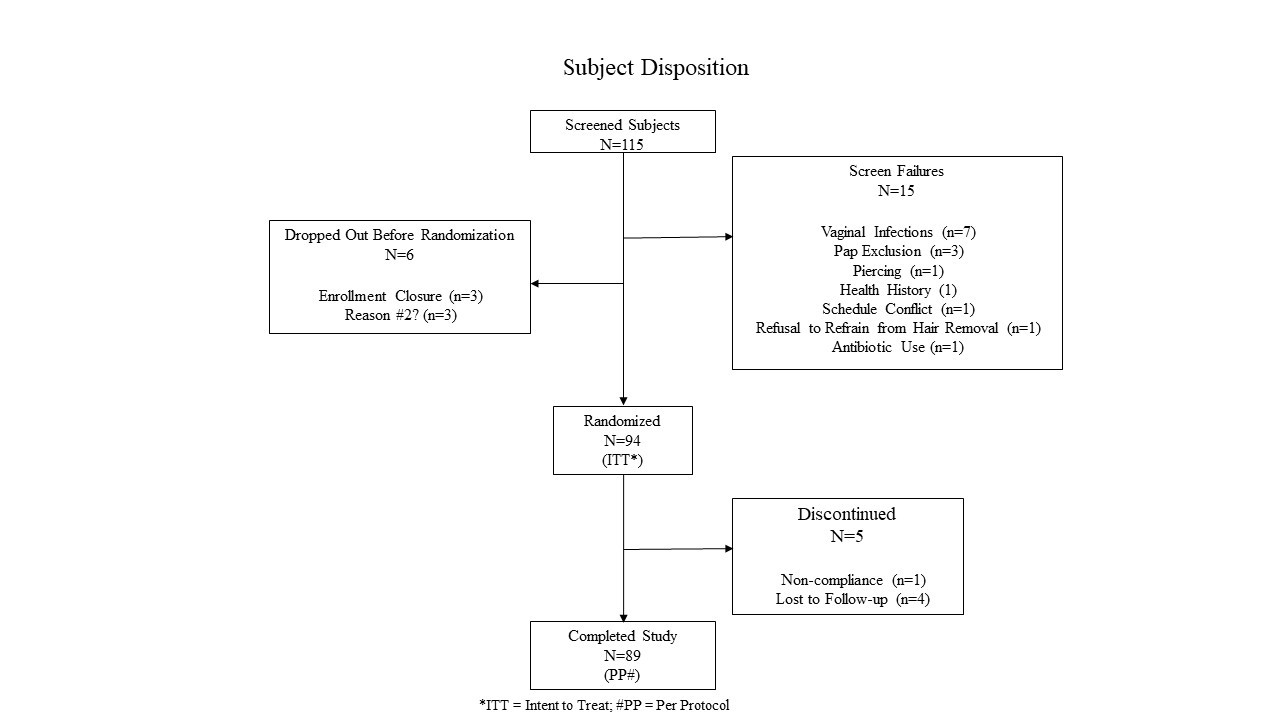

Supplement: Supplementary file 2 [file Image1.jpeg]

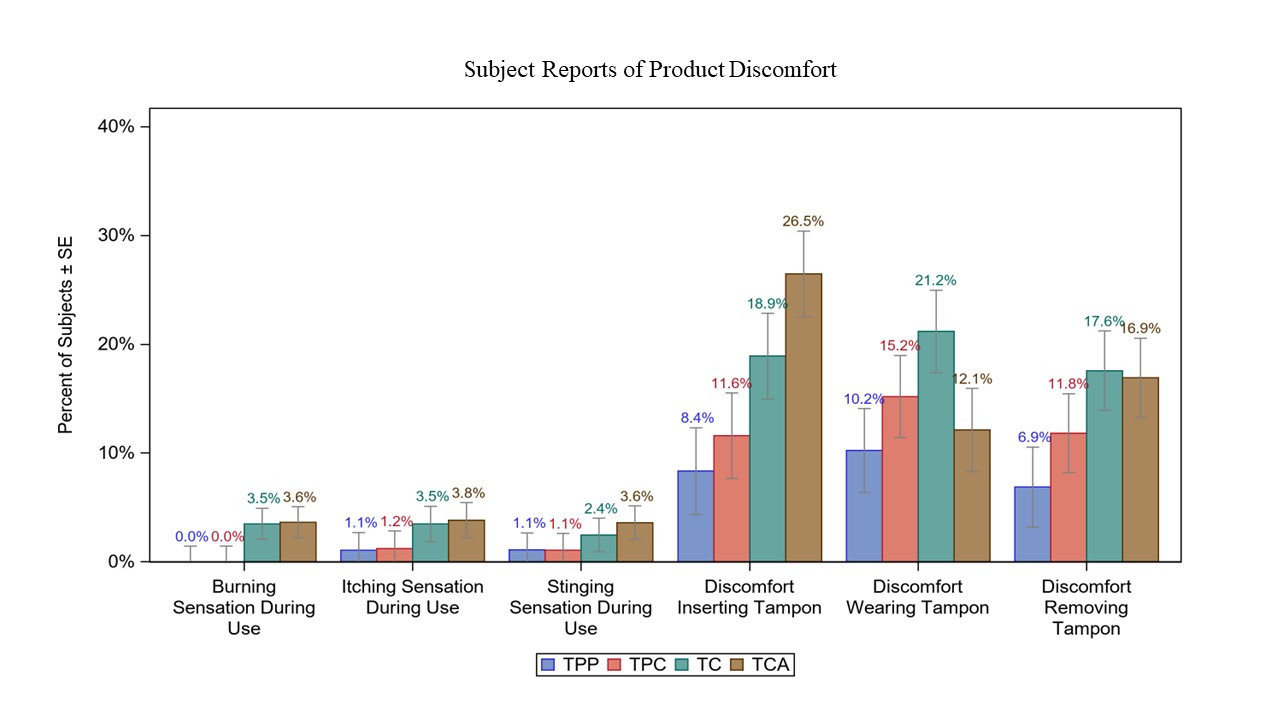

Supplement: Supplementary file 3 [file Image2.jpeg]
